# Improving the value of public RNA-seq expression data by phenotype prediction

**DOI:** 10.1093/nar/gky102

**Published:** 2018-03-05

**Authors:** Shannon E Ellis, Leonardo Collado-Torres, Andrew Jaffe, Jeffrey T Leek

**Affiliations:** 1 Department of Biostatistics, Johns Hopkins Bloomberg School of Public Health, USA; 2 Center for Computational Biology, Johns Hopkins University, USA; 3 Lieber Institute for Brain Development, Johns Hopkins Medical Campus, USA; 4 Department of Mental Health, Johns Hopkins Bloomberg School of Public Health, USA

## Abstract

Publicly available genomic data are a valuable resource for studying normal human variation and disease, but these data are often not well labeled or annotated. The lack of phenotype information for public genomic data severely limits their utility for addressing targeted biological questions. We develop an *in silico phenotyping* approach for predicting critical missing annotation directly from genomic measurements using well-annotated genomic and phenotypic data produced by consortia like TCGA and GTEx as training data. We apply *in silico phenotyping* to a set of 70 000 RNA-seq samples we recently processed on a common pipeline as part of the *recount2* project. We use gene expression data to build and evaluate predictors for both biological phenotypes (sex, tissue, sample source) and experimental conditions (sequencing strategy). We demonstrate how these predictions can be used to study cross-sample properties of public genomic data, select genomic projects with specific characteristics, and perform downstream analyses using predicted phenotypes. The methods to perform phenotype prediction are available in the *phenopredict* R package and the predictions for *recount2* are available from the *recount* R package. With data and phenotype information available for 70,000 human samples, expression data is available for use on a scale that was not previously feasible.

## INTRODUCTION

RNA sequencing (RNA-seq) has become the gold standard for assaying gene expression and has advanced our understanding of transcription (1–3). To date, tens of thousands of samples have been profiled using RNA-seq technology, and their data have been deposited into the public archive (4); however, each individual study typically includes only a small number of samples (average study size for human RNA-seq projects using Illumina sequencing within the sequence read archive (SRA) is 24 samples). Without robust sample sizes, reproducibility across studies has been limited (5–8). Further, while the scientific community has ensured that these data are publicly available, current repository platforms do not make these data easily accessible for future study. Data in public repositories are often not provided in a consistent format, are not annotated clearly for easy use by independent researchers, or have not all been processed using the same methodology, all of which limits comparability across studies. To assist in improving the utility of the available human expression data in the public repository, we previously developed the recount2 (9) resource (https://jhubiostatistics.shinyapps.io/recount/) that includes expression data for 70 000 human samples aligned using Rail-RNA (10). The use of a processing pipeline makes direct comparisons across studies easier. These data, which include expression estimates at the gene, exon, junction, and expressed region levels for each sample, are all available in the recount2 (9) resource and can be easily accessed using the R package recount, dramatically reducing previous barriers to accessibility.

**Figure 1.**
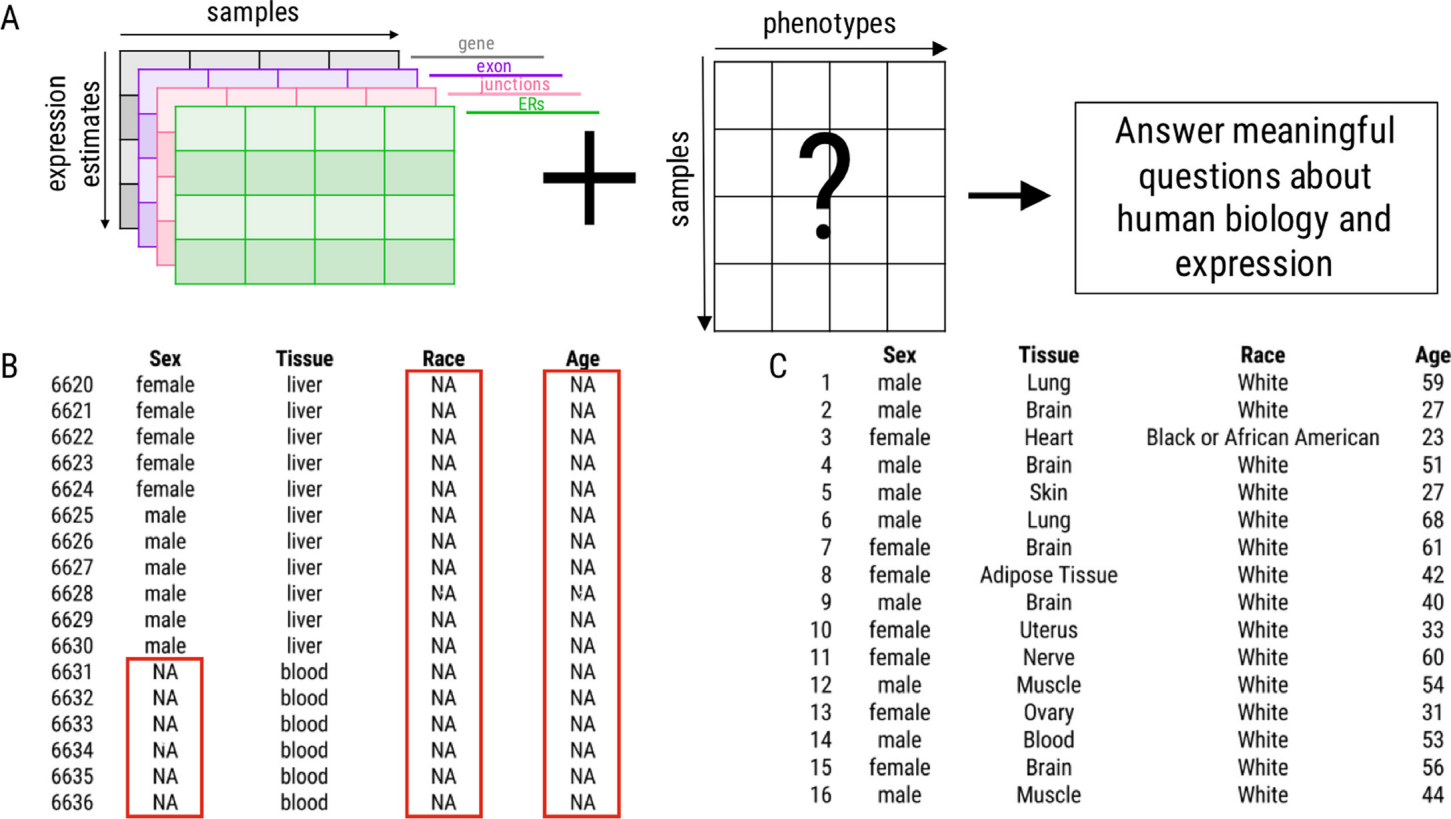
Missing phenotype information. (**A**) Phenotype information is critical to answer questions about biology using expression data. (**B**) This critical information is missing for many samples within the SRA (red boxes). Note that sample phenotype information begins with the 6,620^th^ row, as this is the first row in the dataset for which sex and tissue are available for the same sample. (**C**) Missingness is limited within the GTEx data. Expression data from samples with accompanying phenotype information are used to build the predictors. ERs = expressed regions

While expression information is now available in recount2 (9), for a large number of these samples, phenotype information is not (Figure 1). For example, while there is expression data available from the sequence read archive (SRA) for 49 657 samples, information for the sex of individual from which the sample was taken is only available for 3640 (7.3%) samples. Clearly, without such critical sample information, downstream analytic utility is limited. Further, as these data were initially generated in many different labs, analysis using these data must be concerned about unwanted sources of variation affecting their analyses (11). It is critical to have technical phenotype information available, including information such as sequencing strategy employed, across samples.

**Figure 2.**
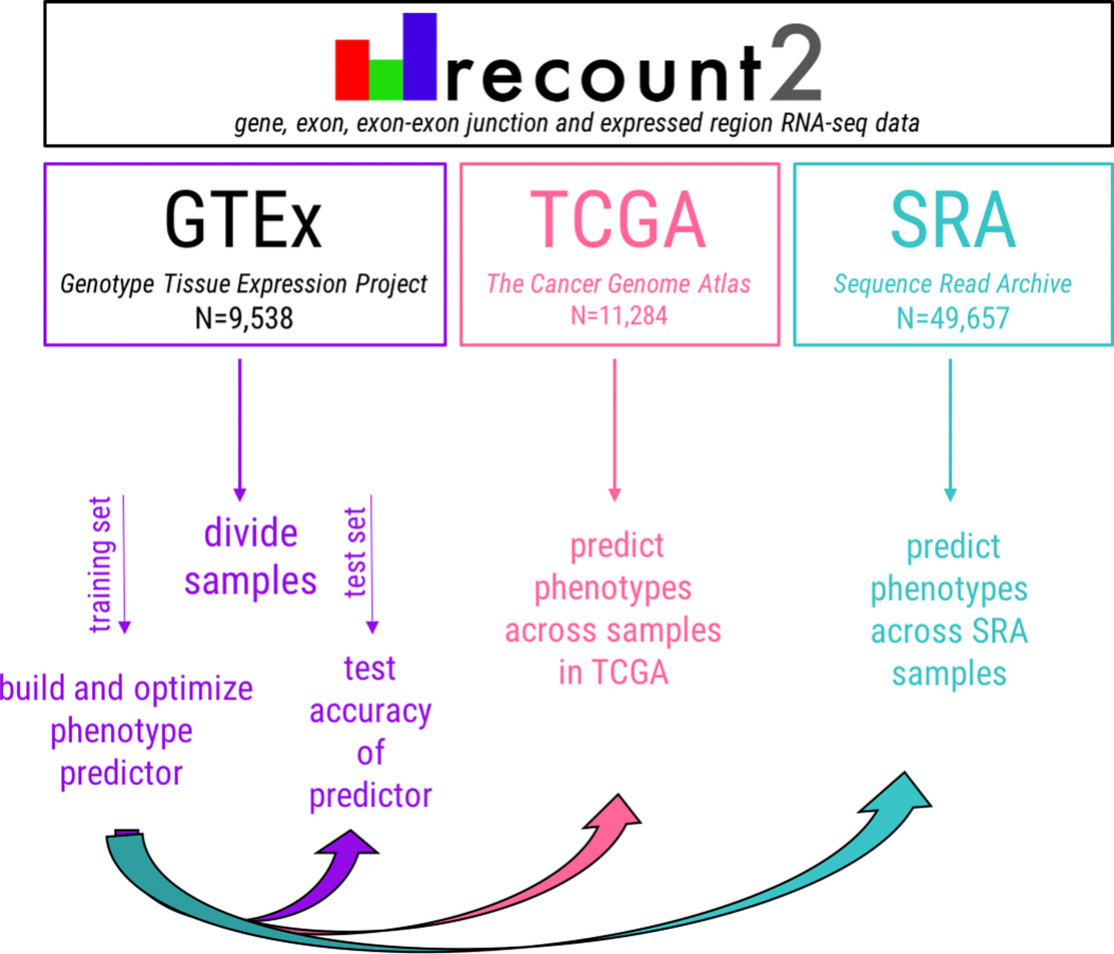
General approach to phenotype prediction. To predict phenotype information, the training data are first randomly divided and the predictor is built. Accuracy is first tested in the training data. Upon achieving sufficient accuracy (≥85%), the predictor is tested in the remaining half of the training data set. Phenotypes can then be predicted across all samples in recount2.

To address this missing phenotype issue, we have built predictors from the expression estimates themselves for a number of biological and technical phenotypes (Figure 2). Using the data within recount2 (9), which includes samples from the Genotype-Tissue Expression Project (GTEx) (12,13) (N=9,538), The Cancer Genome Atlas (TCGA) (http://cancergenome.nih.gov/) (*N* = 11 284), and the sequence read archive (SRA) (*N* = 49 657) (4), we have identified regions that are able to accurately predict (1) sex, (2) sample source (whether the sample was generated from a cell line or tissue sample), (3) tissue and (4) the sequencing strategy (single or paired end) employed. With this critical sample information available for all samples across recount2, in conjunction with the recently-released MetaSRA (14), which provides normalized sample-specific metadata across samples in the SRA, the utility of the data increases such that accurate downstream analyses (i.e. differential expression analyses) are possible.

Finally, we highlight three possible use cases to demonstrate how the phenotypes predicted can be useful in both their own right as well as for use in future analyses. First, there has historically been a sex bias in biomedical research, such that in both humans and in mice, samples from males are utilized more frequently than females (15,16). Accordingly, we demonstrate how predicted phenotypes can be used to characterize the overall breakdown by sex for samples in the SRA. Second, we demonstrate how the phenotypes we have predicted can be used to identify studies whose data may be of particular interest to researchers for their analyses. Last, after identifying a study of interest, we demonstrate how predicted phenotypes can be incorporated into an expression study to gain insight into biology.

## MATERIALS AND METHODS

### Sample summary

Human samples processed using Rail-RNA (10) and incorporated into recount2 (9) were compiled from the v6 release of Genotype-Tissue Expression Project (GTEx) samples, the sequence read archive (SRA), and the Cancer Genome Atlas (TCGA). The SRA data are comprised of 49 657 publicly available samples from 2034 distinct RNA-seq projects. The GTEx samples include RNA-seq data for 9538 samples from 550 individuals and 30 tissues. And, the TCGA samples contributed 11 284 samples from both healthy and cancerous tissue samples. Detailed sample and processing information can be found in Collado-Torres and Nellore *et al.* (9).

### Region level expression data summary

For prediction, as to not limit prediction to annotated regions of the genomes, expressed region (ER) (7,17) level data were utilized. All GTEx samples were normalized to a library size of 40 million 100 base-pair reads to compute the mean coverage, as available from recount2 (9). Expressed regions were defined using a cutoff of 0.5 with the findRegions()derfinder (7) function resulting in 1 187 643 ERs. The coverage matrix for the ERs was computed using version 0.99.16 of the recount.bwtool R package available at https://github.com/LieberInstitute/recount.bwtool to obtain expression estimates at the same ERs identified in the GTEx data. ERs were then log_2_ transformed. recount.bwtool has several scripts for computing the coverage matrices for each project in recount2 using bwtool (18). The function coverage_matrix_bwtool() is similar to coverage_matrix() from the recount package. It is faster and less resource-intensive but requires the recount2 BigWig coverage files be available locally. recount.bwtool is designed for use cases that require computing coverage matrices for a large number of regions and for a large number of samples, such as this one.

### General approach to prediction

The GTEx data are well-characterized and are comprised of data generated from hundreds of individuals, with each individual having been sampled at multiple different tissues. Given the study design and well-characterized nature of these data, when possible (sex, tissue), the GTEx expression data were used to build the phenotype predictors. When invariability of a phenotype within GTEx limited this approach (sample source, sequencing strategy), the SRA data were used to train the predictor. The first step of our general approach was to randomly sample half of the training data set. Expression data from this subset of individuals comprised our training set. For each predictor, accuracy was then assessed in samples from three tests sets: (i) The remaining samples from the training data set that were not used to build the predictor (ii) TCGA and (iii) the data set not used to build the predictor either SRA (in the case of sex and tissue) or GTEx (in the case of sample origin and sequencing strategy). Accuracy was assessed by comparing the predicted phenotype to the reported phenotype in any sample for which this information was available. Upon achieving sufficient prediction accuracy (≥85% in the training data), phenotypes were then predicted across all samples within recount2.

### R package: phenopredict

We have built the R package phenopredict which can use expression data and phenotype information from any study to build a phenotype predictor. Functionality within this package allows for (i) a predictor to be built for either continuous or categorical variables (build_predictor()), (ii) the predictor to be tested on the training data to assess resubstitution error (test_predictor()), (iii) data to be extracted from a new data set for the same regions upon which the predictor was built (extract_data()) and (iv) phenotype prediction in this new data set (predict_pheno()). Additionally, for analyses in which data have to be filtered across multiple files (i.e. across chromosomes), there are two functions (filter_regions() and merge_input()) that can be used to limit the number of regions included for prediction from each file and to merge the filtered regions prior to building the predictor, respectively. Finally, to optimize prediction accuracy, the function optimize_numRegions() can be used to determine the optimal number of regions to be used in build_predictor().

### Building the predictor: build_predictor()

#### Categorical variables

The predictor is built by first employing a linear model within limma’s lmFit() framework (19) to select a set of discriminating regions for each level in the categorical phenotype. In this framework, for each level (*l*) in the phenotype (*P*),

(1) \begin{equation*} { E[\mathbf {E}_r \mid \mathbf {P}_l] = \mathbf {\alpha }_{0r} + {\mathbf {P}_l{\alpha }_{r} }} \end{equation*}

where $\mathbf {P}_l= \left\lbrace \begin{array}{@{}l@{\quad }l@{}}1, & \text{if level of phenotype} \\ 0, & \text{otherwise} \end{array}\right.$ and $\mathbf {E}_r$ is expression at region *r*. The $\mathbf {P}_l$ design matrix minimally contains information about the level *l* of the phenotype of interest (*P*), but it can optionally include other covariates. To ensure predictors herein were as generalizable across data sets as possible, no covariates were included during region selection. $\mathbf {\alpha }_{0r}$ and $\mathbf {\alpha }_{r}$ are the coefficients relating the expression of region *r* to phenotype *P*_*l*_. We use limma (19) to moderate variances across the regions tested, thus shrinking region-wise sample variances. These moderated variances are utilized in identification of regions most highly associated with the phenotype of interest. For each level (*l*), the topTable() function from limma is used to chose the *n* most significant regions. For predictions herein, the number of regions (*n*) was optimized using optimize_numRegions() function within phenopredict. This choses the fewest regions needed to maximize prediction accuracy in the training data; however, users can alternatively specify their desired number of regions for prediction.

After determining the set or regions to be used for prediction ($\mathcal {S}$), validationCellType() from the R package minfi (20) is used to fit the model for each region *r* in the set of regions selected $\mathcal {S}$ using Equation (1):

(2) \begin{equation*} {E[\lbrace \mathbf {E}_r\rbrace _{r \in \mathcal {S}} \mid \mathbf {P}] = \mathbf {\beta }_{r}\mathbf {P}} \end{equation*}

where ($\mathbf {E}$) is expression for every region (*r*) in the selected set or regions ($\mathcal {S}$) and $\mathbf {P}$ is an *l* × *N* matrix where *l* is the number of levels in the phenotype and *N* is the number of samples in the training data. We fit the model subject to the conditions that: (i) $\mathbf {\beta }_{r} >0 \forall \mathbf {\beta }_{r}$, as proposed by Houseman *et al.* (21) and implemented in the minfi (20) package and (ii) no intercept term is included so that $\mathbf {\hat{\beta }}_{r}$ contains the mean expression estimate for each region (*r*) across all levels (*l*) of phenotype ($\mathbf {P}$).

#### Continuous variables

The same approach as above is carried out with the exception that, in Equation (1), we replace the phenotype with a natural cubic spline basis *P*_*l*_ = *s*_*l*_(*P*) (where *l* = 5 and *s*_*l*_ is a natural cubic spline basis function).

(3) \begin{equation*} { E[\mathbf {E}_r \mid \mathbf {P}_l] = \mathbf {\alpha }_{0r} + \sum _{l} {\mathbf {s_l(P)}{\alpha }_{rl} }} \end{equation*}

Selected regions are then modeled as in Equation (2). We fit a single regression model including a spline basis for each of the selected regions. For each region, we set the knots to be equally spaced at the quantiles of all values. Since there is zero inflation among the expression values, sometimes this leads to multiple knots at zero. In these cases we set the first knot at zero, and then distribute the other knots along the quantiles of the non-zero values.

(4) \begin{equation*} {E[\mathbf {P} \mid \lbrace \mathbf {E}_r\rbrace _{r \in \mathcal {S}} ] = \sum _{l,r \in \mathcal {S}} \mathbf {\beta }_{rl}s_l(\mathbf {E_r})} \end{equation*}

### Resubstitution error: test_predictor()

#### Categorical variables

To assess prediction accuracy in the training data, where truth is known, predictions are made relating the coefficients ($\mathbf {\hat{\beta }}_r$) calculated by the build_predictor() function. Here, using the framework established in projectCellType() from minfi (20,21):

(5) \begin{equation*} {E[\lbrace \mathbf {E}_r\rbrace _{r \in \mathcal {S}} \mid \mathbf {\hat{\beta }}_{r} ] = \mathbf {\hat{\beta }}_{r}\mathbf {\gamma }} \end{equation*}

where $\mathbf {\hat{\beta }}_{r}$ are the estimates of mean expression for each level (*l*) in phenotype ($\mathbf {P}$) from Equation (2). $\mathbf {\hat{\gamma }}$ is an *l* × *N* matrix containing the likelihood of belonging to each level in the phenotype. For each sample in *N*, predicted phenotype is subsequently assigned to $max_l(\mathbf {\hat{\gamma }})$. If $max_l(\mathbf {\hat{\gamma }})$ corresponds to more than one level within the phenotype, the sample is predicted as ‘unassigned.’ Finally, this function reports (i) predicted phenotypes, (ii) reported phenotypes (when available) and (iii) resubstitution error (RE), where:

(6) \begin{equation*} \textrm {RE} = \left(1 - \dfrac{ {\# \,\textrm {of samples predicted correctly}}}{ {\#\, \textrm{of samples predicted}}} \right) * 100 \end{equation*}

#### Continuous variables

Predictions are made from model 4 and we measure error as the root mean squared error from the observed phenotype to the predicted phenotype in the training set.

### Extracting regions in a new data set: extract_data()

Before prediction can be carried out in a new data set, expression data at the precise regions used to build the predictor must be extracted. extract_data() can be used to obtain expression estimates in a new data set at the same exact regions selected by filter_regions() and used to build the predictor. By using expression from a fixed set of genomic regions in the new data set, rather than identifying a new set of predictive regions, we limit the possibility of overfitting our model.

### Phenotype prediction: predict_pheno()

#### Categorical variables

Phenotype prediction in a new data set occurs as in Equation (5) of test_predictor() with the exception that expression data in this case comes from a new expression data set ($\mathbf {E}_r^*$), which contains expression information for the same set of regions (*r*) upon which the predictor was built, but from an independent set of samples (N*), such that:

(7) \begin{equation*} {E[\lbrace \mathbf {E}_r^*\rbrace _{r \in \mathcal {S}} \mid \mathbf {\hat{\beta }}_{r} ] = \mathbf {\hat{\beta }}_{r}\mathbf {\gamma }^*} \end{equation*}

Here, $\mathbf {E}_r^*$ contains the expression estimates at the same set or regions used in Equation (2), but for the samples in this new data set (N*). $\mathbf {\hat{\beta }}_{r}$ remains the estimates of mean expression for each level (*l*) in phenotype *P*) from Equation (2). Here, however, $\mathbf {\gamma }^*$ is an *l* × N* matrix containing the likelihood of belonging to each level in the phenotype for this new set of samples. Phenotype is assigned as described in test_prediction().

#### Continuous variables

Predictions are made from model 4 and we measure error as the root mean squared error from the observed phenotype to the predicted phenotype using the new expression data ($\mathbf {E}_r^*$) as the predictors.

### Assessing accuracy

#### Categorical variables

The subset of samples within each data set for which reported phenotypes were available were used to assess accuracy, such that:

(8) \begin{equation*} \textrm {Accuracy} = \dfrac{{\#\, \textrm {of samples predicted correctly}}}{ {\#\, \textrm {of samples predicted}}}*100 \end{equation*}

Here, the number of samples predicted includes all samples for which phenotype information was available and whose reported value was included in our predictor (see Supplementary Table S1). Using sex as an example, all SRA samples whose sex was reported as ‘male’, ‘M’, ‘Male’, ‘m’, ‘female’, ‘F’, ‘Female’ or ‘f’ were included in the denominator. However, samples whose reported sex was missing (NA) or whose reported value was not meaningful (i.e.‘not collected’, ‘not determined’, ‘Unknown’, etc.) were not included in the calculation of accuracy. Sex was, however, predicted across all samples after accuracy assessment.

#### Continuous variables

Accuracy was again assessed using the correlation coefficient (*R*^2^) and RMSE between the reported and predicted values, for samples where information was available.

#### Sensitivity and specificity

For all phenotypes included in recount2, accuracy was further assessed by constructing confusion matrices, to explicitly demonstrate what phenotypes were predicted when predictions were different from what was in the reference. Further, sensitivity and specificity were both calculated for every level of each predicted phenotype. Sensitivity was defined such that *Sensitivity* = TP/(TP + FN), where TP are the number of true positives and FN are the number of false negatives. Specificity was defined such that Specificity = TN/(TN + FP), where TN are true negatives and FP are false positives.

### Integration with recount2

Predicted phenotypes are available through the R package recount. Predicted phenotypes can be included using the add_predictions() function. For each predicted phenotype, this function adds reported phenotype, predicted phenotype, and prediction accuracy. The most recent release will be used by default; however, older versions will remain accessible through recount. An example of this function is provided at https://gist.github.com/ShanEllis/?add_pred.R and demonstrates how to add predicted phenotypes to the GTEx data within recount.

### Using an established predictor for prediction in a new data set

To predict sex, age, sequencing strategy, or sample source in a new data set using the predictors presented herein, users would first obtain the predictor data using the following: data(prebuilt_predictors, package=‘phenopredict’). This imports the prediction coefficients and region information for each of the four predictors: predictor_sex, predictor_tissue, predictor_samplesource, and predictor_sequencing strategy. The predictor object for the phenotype the user is interested in predicting (i.e. ‘predictor_sex’ if interested in predicting sex) would then be used as the predictordata argument in the extract_data() function within phenopredict.

### Use case #1: Sex analysis within the SRA

Sex within the SRA was studied across the 49,657 samples in recount2. Predicted sex was first summarized overall, where each sample received a predicted sex of male, female, or unassigned (when sex could not be disambiguated). Secondarily, sex was summarized by project. When all the samples within a project were of the same predicted sex, they were classified accordingly as female only, male only, or unassigned only; however, projects with samples of more than one predicted sex were determined to be ‘mixed’. Finally, sample size broken down by sex within each project type was summarized.

### Use case #2: Using predicted phenotypes to identify studies of interest

Predicted phenotypes can be used to identify samples or studies of interest for future study. To exemplify this, constraints were placed on the predicted phenotype data to search for a study within the cancer studies in recount2 requiring that possible projects must (i) employ paired RNA-seq, (ii) not have reported sex included in the study metadata, (iii) have ‘tissue’ as its predicted sample source (rather than be derived from a cell line) and (iv) have at least 20 samples in the study. Studies fitting these criteria were considered for use in Use case #3.

### Use case #3: Using predicted phenotypes in analyses

After identifying three studies in case study #2 that fit our criteria (‘SRP029880’, ‘SRP027530’, ‘SRP055438’), the corresponding abstracts for each study were searched within recount2 (9). It became immediately clear that SRP055438 is a Crohn’s study in which some patients had cancer, rather than a cancer study, and was thus excluded. Between the other two studies, ‘SRP027530’ had a smaller sample size (*N*_*SRP*027530_ = 20; *N*_*SRP*029880_ = 54) and the abstract of this study included the breakdown of females and male samples (22). Thus, despite the fact that sex was not directly reported in or accessible from the SRA metadata, a quick search made it clear that sex could be relatively easily obtained for this study. As we were interested in studying the effects of sex on an analysis that otherwise did not include sex in their analysis, we moved to study SRP029880.

Gene-level count data were downloaded from recount2 (9) for project SRP029880. Raw counts were scaled to the total coverage of the sample. Cancer status and sample id were obtained from the reported SRA metadata field ‘title’. Differential gene expression analysis was first carried out as reported in the initial publication (23). Briefly here, DGEA was carried out using the edgeR (24) R package, which assumes a negative binomial distribution for expression to detect differentially expressed genes. Both normal colon epithelium (NC) and liver metastases (MC) were compared to primary colorectal tissue (PC) samples. To recapitulate the analysis from the initial publication, no outlier genes or samples were removed, no covariates were included, and no adjustment was made for the fact that the PC samples were used in both comparisons. As was done in the initial publication, to protect against hypervariable genes driving differences detected, gene dispersion was estimated for the scaled gene counts for 58 037 genes across 54 samples (18 unique individuals). For each gene (using the estimated dispersions calculated), and each comparison (NC:PC, MC:PC) a negative binomial generalized log-linear model (glm) was fit. To be in line with the initial publication (23), genes were considered statistically significant if the p-value was <0.0001 and the log fold change (logFC) was ≥2. Finally, in Kim *et al.* (23) ‘liver-specific genes’ were removed from the MC:PC comparison in hopes of accounting for the different tissue source of the two sets of samples. The initial publication noted 309 genes were removed; however, a list with 383 genes was referenced. As the 309 genes list was not published, this list of 383 genes (25) was used to remove genes from the MC:PC analyses herein.

Liver (*N* = 136) and colon (*N* = 376) samples from the GTEx data were utilized for further comparison. GTEx gene expression data (‘SRP012682’) were downloaded from recount2 (9). Count data were scaled, and genes with fewer than 5 reads average across samples were removed from analysis. Differential gene expression analysis between liver and colon for these 29,966 genes was carried out as described above. Liver-specific genes, as defined above, were again removed from analysis. Concordance at the top (CAT) plots comparing genes differentially expressed (*P*-value < 0.0001 and logFC ≥ 2) between GTEx liver and colon samples and the results from both the NC:PC and MC:PC comparisons were generated.

### Availability of data and materials

Analyses were all carried out in R 3.4.1. Code for the R package phenopredict is available at https://github.com/leekgroup/phenopredict. Code for prediction of each phenotype is available at https://github.com/ShanEllis/phenopredict_phenotypes. Use case code is available at https://github.com/ShanEllis/phenopredict_usecase. The R package recount.bwtool to extract region level data from samples in recount2 is available at https://github.com/LieberInstitute/recount.bwtool. The GitHub repositories that contain code used in this manuscript can be viewed at the precise version that was used at time of publication. The full code for these repositories can be downloaded via GitHub using the particular commit versions in Supplementary Table S8.

### List of abbreviations

RNA-Sequencing (RNA-seq); Genotype Tissue Expression Project (GTEx); The Cancer Genome Atlas (TCGA); Sequence Read Archive (SRA); expressed region (ER); generalized log-linear model (glm); log fold change (logFC); colorectal cancer (CRC); normal colon epithelium (NC); liver metastases (MC); primary colorectal tissue (PC); Differential gene expression analysis (DGEA); Root mean square error (RMSE); concordance at the top (CAT).

## RESULTS AND DISCUSSION

### Phenotype prediction

We harnessed the inherent variability of expression data to accurately predict important biological (i.e. sex, tissue, sample source) and technical (i.e. sequencing strategy) phenotypes from the expression data itself. All phenotype predictors were built using the data available in recount2 (9). Data included within this resource include RNA-seq data from (i) the GTEx Project (*N* = 9538), TCGA (*N* = 11 284) and the SRA (*N* = 49 657). Region-level expression estimates (7,17), which are annotation-agnostic, were used for prediction. To build the predictors included here and to enable future predictors to be built using this approach, we have developed the R package phenopredict, which includes all the necessary functions to build and test predictors from expression data. Number of both samples used and regions included for predictions included herein are summarized in Supplementary Table S1.

### Regions used for prediction

For categorical variables, speed of building each predictor is dependent upon both the number of levels in the categorical variable and the number of regions used for prediction. Thus, the number of regions used for prediction has to be minimized while maintaining prediction accuracy. To optimize the number of regions included for prediction, eight values (ranging between 10 and 200) for the numRegions argument were tested for each predictor. For categorical variables, numRegions determines how many regions should be selected for prediction from each level in the phenotype; thus, the total number of regions can be up to *numRegions* × *levels*. The minimum numRegions value that maximized accuracy in the training set was then utilized for phenotype prediction. This corresponded to the numRegions argument being equal to 20 for both sex and sequencing strategy (resulting in 40 total regions used for prediction), 100 for sample source (200 total regions), and 80 for tissue (2281 total regions) (Supplementary Figure S1).

To characterize these regions further, the genomic location for each region was summarized. Aside from sex, where regions were intentionally only selected from the sex chromosomes, no single chromosome contributed >17.5% of the regions used in any predictor (Supplementary Figure S2A). Additionally, selected regions spanned the length of the chromosomes, demonstrating no obvious regional bias for prediction (Supplementary Figure S2B). Taken together, this demonstrates that for each of the predictors, regions across the genome are contributing to prediction. Finally, to provide functional context, each region was assigned to a genomic category. As these data are expression data, it can be expected that most regions will overlap with annotated genes. Among the regions used, depending upon the phenotype, between 67.5% and 85.9%, overlapped with annotated genes (including the 5′, exons, and 3′ regions of the gene). However, as the underlying data here are expressed regions, expression outside of annotated exons is not surprising. Further, due to the fact that differential expression at genes likely drives prediction for biological phenotypes (sex, tissue, and sample source), we hypothesized that a higher proportion of regions for these phenotypes would overlap with annotated genes while regions for technical phenotypes (such as sequencing strategy) may fall more frequently outside of annotated genes, reflective of expression differences due to technical artifacts. Here, 32.5% of the regions for sequencing strategy fall outside annotated exons relative to 14.1%, 15.0% and 19.5% for tissue, sex, and sample source, respectively, supporting the hypothesis.

### Sex prediction

**Figure 3.**
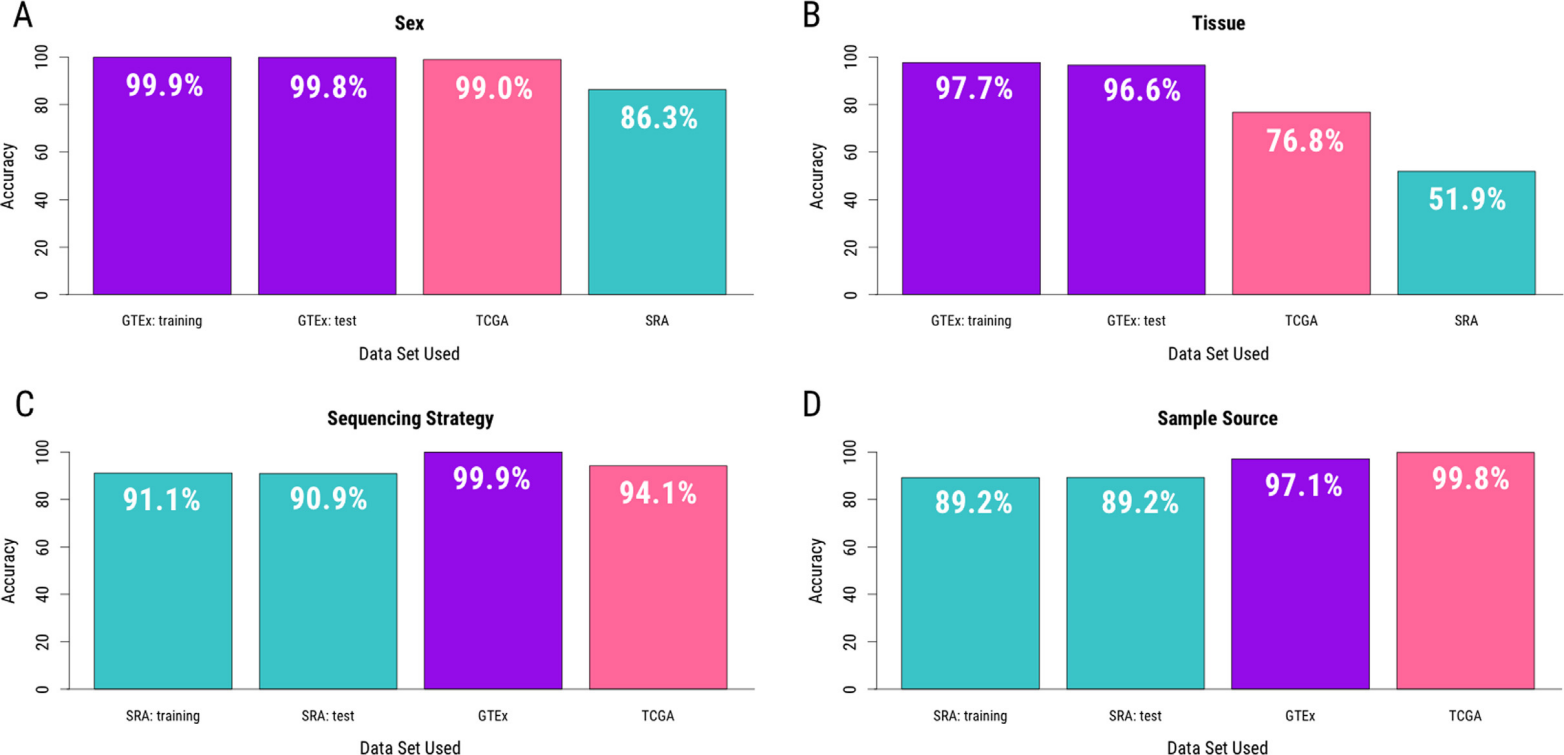
Prediction accuracy. Predictors for critical phenotype information were built from expression data available in recount2 for (**A**) sex, (**B**) tissue, (**C**) sequencing strategy and (**D**) sample source. Samples for which reported phenotype information is available were used to determine prediction accuracy. GTEx data are in purple, TCGA in pink, and SRA in teal.

Sex prediction from RNA-seq data benefits from the biological fact that males and females differ in their sex chromosome composition. To build a predictor for sex from expression data, the GTEx data were first split into a training set (*N* = 4769) and a test set (*N* = 4769). The 40 regions (20 each from the X and Y chromosomes) that best discriminated males and females in the GTEx training data were selected and the predictor built. In the GTEx training data, when applying the predictor to the data upon which it was trained, the resubstitution error (see Materials and Methods section) was 0.1%. Sex prediction was then carried out in the GTEx test set, an expression data set in which the data were generated by the same consortia but whose samples were not included to build the predictor. Prediction accuracy was 99.8% in these data. Sex prediction was carried out in data from TCGA (*N* = 11 284), a completely independent and well-characterized data set, where prediction accuracy was 99.0%, and in the SRA samples for which sex information was available (*N* = 3640), where accuracy was 86.3% (Figure 3A).

A decrease in accuracy within the SRA was expected due to the heterogeneity of the SRA data; however, to investigate this drop in accuracy more closely, the study-wise accuracy for all SRA studies where sex was reported (3640 samples across 211 studies) was determined. Median study accuracy was 100% (range: 0–100%), with 72% of studies having a prediction accuracy >90% (Supplementary Figure S3A). One relatively large study (’SRP040547’, *N* = 200) demonstrated notably low accuracy (1.14%) (Supplementary Figure S3B). Upon closer inspection, sex was reported to be ‘male’ in 88 samples and ‘N/A’ in the remaining 112; however, only 44 of the 200 samples were predicted to be ‘male’ and only one of these 44 samples predicted to be male overlapped with the samples originally reported to be male. This 2014 study looked to experimentally infect human host cells with varying *Toxoplasma gondii* parasite lineages to generate expression profiles. Discordant predicted and reported sex possibly suggests that infection altered expression to such an extent on the X and Y chromosomes that accurate sex prediction was impossible; however, an alternate hypothesis could be that sex was incorrectly reported in the SRA metadata. To investigate this possibility, gene expression data from the Y chromosome for this study were obtained from recount2. Twenty-four genes with mean count across samples >100 were included for analysis. Median expression across these genes for each sample was subsequently calculated. We hypothesized that male samples should show high expression at these genes, while females, who lack a Y chromosome should not. Median log_2_(expression) was zero in both samples reported to be male and those samples predicted to be female, while samples predicted to be male had a median log_2_(expression) of 6.3 (Supplementary Figure S4A). These results suggest that misreporting in the SRA metadata is likely. Removing this study from accuracy calculation only improves prediction accuracy by 2.1% to 88.4%, demonstrating that no one study is solely responsible for the decreased sex prediction accuracy across the SRA. To assess if misreported sex was widespread in studies with low sex prediction accuracy, we carried out the same analysis in all studies with a prediction accuracy <70%, with at least 25 samples, and with at least ten genes with mean count >100. This analysis demonstrated that sex is frequently misreported in SRA studies with low sex prediction accuracy (Supplementary Figure S4B–D). Taken together, these analyses support the hypothesis that sex prediction accuracy is likely lower across the SRA due to misreporting in the metadata and that the reported accuracy for sex prediction across the SRA (86.3%) herein is likely a conservative estimate.

### Tissue prediction

Tissue prediction is more complex than sex prediction for two reasons: (i) region selection must be carried out across all chromosomes (not just the sex chromosomes) and (ii) there are more than two levels for which a predictor must be built. In the case of the data in recount2 (9), the GTEx training data include information for 30 different tissues. For each tissue, regions with distinct expression profiles relative to all the other tissues in the data set were selected for inclusion in the predictor. For these analyses, 2281 regions were selected across the autosomes and the sex chromosomes. Accuracy was assessed as above for the GTEx and TCGA data: using the percentage of samples whose tissues correctly predicted from their expression data as our metric. Here, tissue prediction was correct in the GTEx training set 97.7% of the time (2.3% re-substitution error). Accuracy for tissue prediction was 96.6% in the GTEx test set (*N* = 4769) and 76.8% in the TCGA data (*N* = 7317) (Figure 3B). We note, however, that TCGA is a cancer data set that includes samples from cancerous tissues as well as healthy control tissue. When this analysis was stratified to separate healthy tissue samples from cancer tissue samples, prediction was accurate 92.7% of the time in healthy tissue (N=613) and 75.3% of the time in cancerous tissue (*N* = 6704) (Supplementary Figure S5), suggesting that altered expression within cancerous tissue samples may benefit from a separate tissue predictor. Assessment in SRA was less direct as tissue information was infrequently available in the metadata available from the SRA. Instead, to find a metric to which we could compare our predicted tissue types in SRA, a natural language processing tool, sharq (http://www.cs.cmu.edu/∼ckingsf/sharq/) was employed. sharq was previously used to scrape the abstracts for a subset of studies included in the SRA, extracting its best guess as to the tissue used for study from each. Using this tool, tissue type was assigned to 8951 SRA samples. These predictions (‘sharq_beta’) were previously made available in recount2 (9). Comparison between the sharq_beta predictions and our tissue predictions from expression data were concordant in 51.9% of the SRA samples (Figure 3B). By using NLP to predict tissue, accuracy within the SRA is likely a lower bound, and one could expect to see improved accuracy, had reported tissue been directly available for comparison.

To assess this claim directly, we compared predictions from the expression data and sharq_beta predictions to reported phenotypes for the subset of SRA samples where tissue was reported (*N* = 10 830). Among these samples, there were 336 unique reported tissues within the SRA with 1442 (13.3%) samples directly matching the predicted tissue from the expression data and sharq_beta predictions, respectively. This relatively low concordance often reflects nonuniform or distinct coding within the SRA. Specifically, there are 1692 cases in which sharq_beta and expression predictions are concordant with one another but discordant with what is reported in the SRA. Of these, 1131 samples (65.2%) include the predicted tissue in the reported tissue (i.e. predicted tissue is ‘brain’; reported tissue is ‘brain mixed human brain’). Of the remaining 561 samples (561/1131, 49.6%) concordant between sharq_beta and expression predictions but discordant with reported tissue, there are a number of explanations. To assess what could be going on, we randomly sampled one sample from each of the 32 unique studies from which these 561 samples were generated. Of these 32 samples, (i) 62.5% of the time (20/32) the predicted tissue was a more general term for the reported tissue (i.e. predicted tissue is brain; reported tissue is ‘dorsolateral prefrontal cortex’), (ii) 15.6% (5/32) of the time the reported tissue was concordant with the predicted tissue, but the reported tissue also included diagnosis (predicted tissue is ‘adrenal gland’; reported tissue is ‘adrenal tumor’), (iii) 9.3% of the time (3/32) the tissue reported did not indicate an actual tissue (predicted tissue is ‘breast’ but the reported tissue is ‘tumor’), or (iv) 12.5% of the time (4/32) the predictions are discordant (i.e. predicted tissue is ‘liver’; reported tissue is ‘brain’). Taken together these data suggest that true accuracy is likely between the lower bound of 51.9% determined by comparison to sharq_beta predictions and the 87.5% accuracy found in this less rigid comparison to the subset of SRA samples with defined tissue annotations.

### Sequencing strategy prediction

Technical sequencing information, such as whether single or paired end sequencing was carried out, is important information for accurate downstream analysis. To determine whether technical sequencing information could be predicted from expression data, we set out to predict sequencing strategy across recount2. This is necessary within the recount2 resource, as, due to space limitations, raw .bam files are not stored during alignment (9). As one cannot look to the file structure of the raw data simply for confirmation of sequencing strategy, there is further utility, beyond simply being a proof of principle, in being able to recover this information from the expression estimates itself. As the GTEx and TCGA data were all sequenced using a single sequencing strategy, they could not be used to generate the predictor. Instead, to predict this phenotype, the SRA data were split randomly, without stratifying by study or phenotype of interest, into a training set (*N* = 24 829) and a test set (*N* = 24 828). Forty regions that distinguished single end sequencing libraries from paired end sequencing libraries were identified and the predictor built. In the training set, the resubstitution error was 8.9%. Accuracy was then assessed in the SRA test samples (*N* = 24 828), GTEx (9538) and TCGA (*N* = 11 284) samples, where accuracy for sequencing strategy prediction was 90.9%, 99.9%, and 94.1%, respectively (Figure 3C).

### Sample source prediction

To utilize data from a human RNA-seq experiment, it is critical the analyst be aware whether the data were generated from tissue directly or from a cell line. To predict this phenotype from the expression data as with the sequencing strategy predictor, regions were selected from the SRA training set. Within this training set, 10 777 samples for which available metadata clearly stated whether the sample came from a cell line (*N* = 4837; 44.9%) or a primary tissue (*N* = 5940; 55.1%) were used to select 200 regions upon which the predictor was built. The resubstitution error for this predictor was 10.8%. To assess accuracy in the SRA test set samples, all samples for which sample source information were utilized. Prediction accuracy was 89.2% in these data. Applying this predictor across the GTEx (*N* = 9538) and TCGA (*N* = 11 284) data demonstrated its accuracy to be 97.0% and 99.8%, respectively (Figure 3D). While the error rates within the SRA training and test sets are higher than the sex and tissue predictors, this is unsurprising, as this predictor was built in the more heterogeneous SRA samples, where errors in reported phenotypes are more likely (see ‘Sources of prediction error’) and expression data were generated across a number of labs.

### Continuous variable prediction

In an attempt to predict age, 900 regions associated with age were selected from the GTEx training data. While predicted and actual age were correlated in the training data (*R*^2^ = 0.96), the lack of correlation in the test data (*R*^2^ = 9.9 × 10^−3^) suggested limited predictability of this phenotype from expression data (Supplementary Figure S6). This initial attempt to predict age was carried out agnostic to sample tissue of origin. To alternatively test if age could be predicted within tissue, regions associated with age were identified across a number of tissues. Given the limited strength of this relationship (Supplementary Figure S7), we did not pursue prediction of age from expression data by this approach further. Subsequently, prediction for BMI and height were predicted using the same approach but using only the 900 regions for each phenotype that optimized prediction in the training data. Results again showed limited predictably of these phenotypes from expression data. As such, age, BMI and height predictions have not been included for use. The results from these analyses are available, however, on GitHub (https://github.com/ShanEllis/phenopredict_phenotypes). Given these results, some phenotypes will likely be better identified through other means—either by manually curating SRA metadata (14) or through natural language processing (26).

### Variance in prediction across data sets

This approach to prediction utilizes simple modeling and interpretable predictors; however, the trade-off for simplicity is that extreme expression estimates in future data sets could pose a problem for accurate prediction. To assess this as a potential issue, we calculated the variance for each of the 40 regions used for sex prediction across each data set as a test case. Two patterns are discernible from the variance across data sets for these 40 regions (Supplementary Figure S8). First, we note that there is generally more variance within the TCGA and SRA data relative to the GTEx samples. As the TCGA samples contain cancer expression data and the SRA samples are extremely heterogeneous, both of which are expected to increase variance in expression estimates, this result is unsurprising. Secondarily, across the 40 sex prediction regions, expression variance is largely similar across data sets at regions used for sex prediction, suggesting that extreme outliers is largely not an issue. However, there are a few regions that demonstrate increased variance in the TCGA and SRA data. Given the overall sex prediction accuracy, this finding suggests that even when more extreme variance is seen within a data set, it does not prohibit prediction. While more extreme variance does not largely affect accuracy within sex prediction, a predictor which directly addresses or controls for these extreme values at the sample level may see improved accuracy.

### Assessing discordance

For each predicted phenotype, we report prediction accuracies for samples within each dataset (GTEx, TCGA, SRA). By including information about the accuracy of our predictions we make it possible for downstream models to incorporate this uncertainty. While the overall accuracy for any of the predicted phenotypes assessed was at minimum 85%, assessing phenotype accuracy at the sample level is non-trivial. When available, we have included reported phenotype and utilized it to calculate sensitivity and specificity, but this information is not available for many samples and even when it is available, it may be incomplete or incorrect. Phenotype files often use manual data entry and can themselves be prone to error (27). For example, when we look at the 15 GTEx samples whose reported sex and predicted sex disagree (Supplementary Table S2), 13 of these samples come from the same individual (‘GTEX-11ILO’). Further, no samples from this individual had a predicted sex concordant with the sample’s reported sex. Together, this may mean that this sample has mislabeled sex information. The remaining two samples (‘SRR809065’ and ‘SRR658331’), each have 12 other tissue samples from the same subject. In both cases, the other 12 samples were predicted to be the reported sex, supporting that these two samples are either incorrect predictions or mislabeled samples that should likely not be used for further analyses.

To assess discordance levels globally, sensitivity and specificity were calculated for every level of each predicted phenotype. Across predictions, mean sensitivity was 78.6% (range: 18.6–99.1%) and mean specificity was 98.9% (range: 97.2–99.7%) (Supplementary Table S3). Upon closer inspection, confusion matrices (Supplementary Tables S4–S7) demonstrate that these are likely conservative metrics for tissue prediction, as inaccurate predictions are frequently made to biologically similar tissues (Supplementary Figures S9–S11). For example, ‘Fallopian Tube’ was often predicted when the reported tissue was ‘Ovary’ or ‘Adipose Tissue’ was predicted when the reported tissue was ‘Breast’ (Supplementary Figures S9–S11).

### Sources of prediction error

We have reported measures of accuracy for each predictor; however, both reporting error and prediction error affect this measure. Prediction error is error attributable to true limitations of our predictors. This is what we have aimed to minimize in the construction of each of our predictors. Reporting error is error attributable to mislabeling or misreporting within the metadata provided with each data set.

To quantify the level of reporting error directly, the sequencing strategy phenotype was used. Unlike biological phenotypes, where the truth is not always known, for sequencing strategy, a technical phenotype, whether the type of reads in the archive (the truth) matched the strategy reported within the SRA metadata could be used to estimate the level of reporting error within recount. The reads counted by Rail-RNA (10) were compared to the number of spots reported in the SRA to obtain ‘the truth’. If a sample was reported to be ‘single end’ sequencing but the counted reads value exceeded the number of spots reported, the sample was determined to have a misreported ‘single end’ sequencing strategy when it should have been ‘paired end’. Conversely, for samples labeled paired end where there were exactly twice the number of spots as reads counted, this was determined to be a case where a sample was misreported as ‘paired end’ when it should have been ‘single end’. Specifically, there were 207 samples (207/49 182; 0.42%) from 24 unique projects (24/2034; 1.18%) whose reported sequencing strategy in the SRA metadata was independently determined to have been misreported within the SRA. This suggests that sequencing strategy was misreported approximately 1% of the time within the SRA.

In the context of the predictions for sequencing strategy made from the expression data, in the SRA training data, of the 224 samples determined to likely be misreported, 207 (92.4%) were also identified as discordant in the expression predictions, suggesting that the true accuracy of sequencing strategy prediction is 92.0% (22834/24828), rather than the reported 91.1%. Similarly, in the SRA test data, 239 of the 251 (95.2%) samples determined to have been misreported were also discordant between reported and predicted sequencing strategy, suggesting that the true accuracy of sequencing strategy prediction is 92.8% (22798/24829), rather than the 90.9% reported accuracy. This reporting error negatively affects our accuracy measure, further supporting that the accuracy measures we have are reported are conservative, lower-bounds for accuracy.

### Use case #1: sex analysis within the SRA

**Figure 4.**
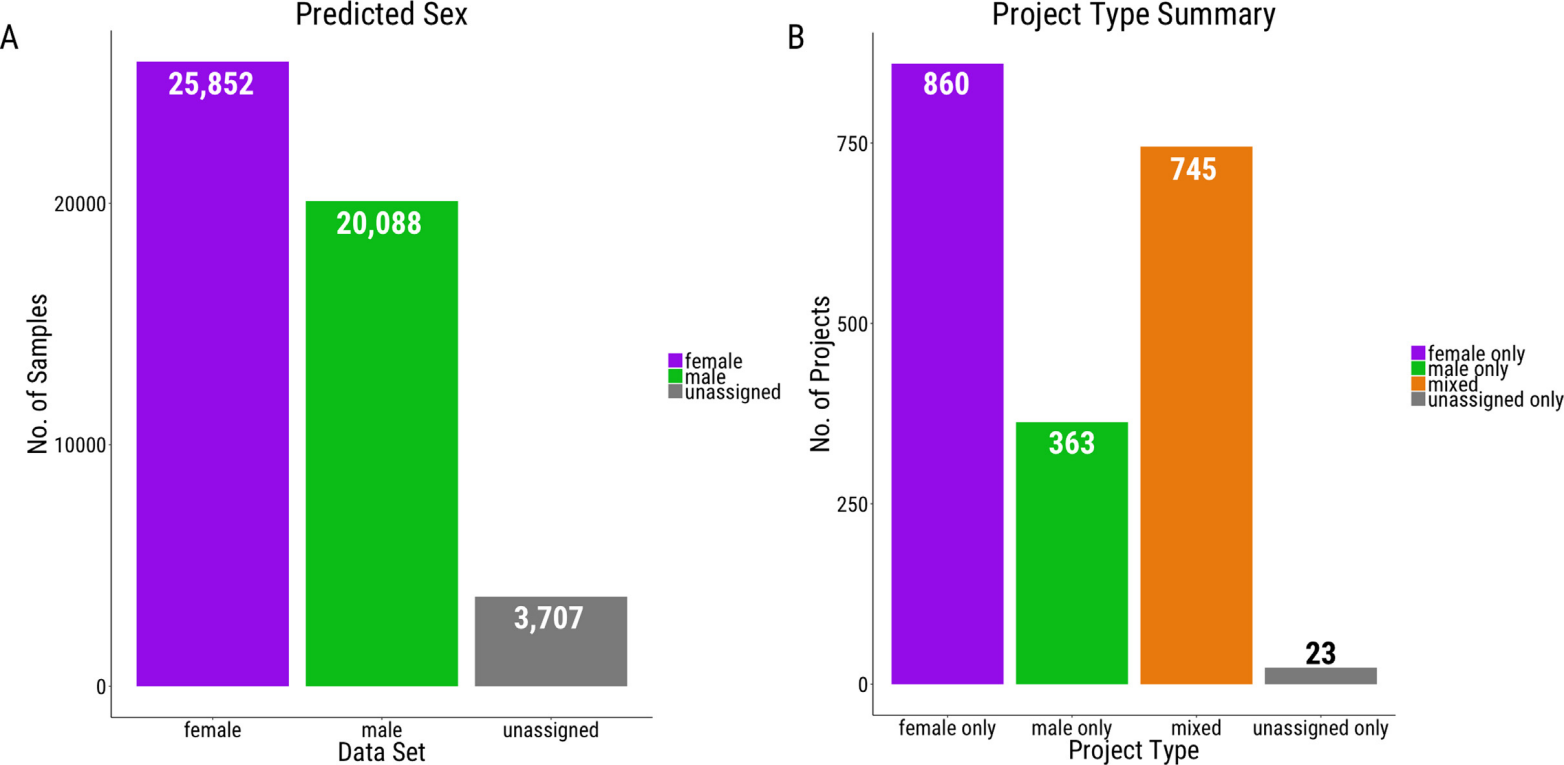
Predicted sex across the SRA Plots summarize predicted sex across the SRA showing (**A**) the distribution of predicted sex across SRA samples, and (**B**) the distribution of project type, broken down by the predicted sex of samples in each project.

There is a long history within the biomedical sciences favoring the study of males relative to females (15,16). To assess the prevalence of this bias within the SRA, we have analyzed the overall breakdown of sex in a number of ways. First, we report that overall within the SRA, 52.1% of the samples are predicted to be female, 40.4% are predicted to be male, and 7.4% could not be disambiguated, suggesting that there is no evidence for a male sex bias within the SRA (Figure 4A). However, one important caveat is that we do not yet have a handle on how many replicate samples there are within the SRA. Looking specifically at a large study within the SRA (SRP025982, *N* = 1720) (28), it becomes clear that this study, in assessing the accuracy and reproducibility of RNA-Seq across platforms and labs, analyzed the same samples, which happened to be female, many times. While this is a clear example of a case where replicate samples are biasing the sex breakdown within SRA, we do not yet have an estimate for how widespread this is across all samples within recount2. Having an idea of the number of repeated measures for each individual within the SRA in the future will help to improve understanding of the of overall breakdown of sex across the SRA.

When broken down by project type, it becomes clear that almost half of the projects within the SRA (43.1%) include exclusively female samples. Of the remaining projects, 18.2% include exclusively male samples, 1.2% include only samples that could not be disambiguated, and 37.4% of studies include some combination of predicted sexes (Figure 4B). For projects where predicted sex includes samples that are either female and unassigned or male and unassigned, one could reasonably assume that in many of these cases, all samples were likely of one biological sex; however, we will leave it up to future investigators to decide if this is a fair assumption to make for their purposes.

### Use case #2: Using predicted phenotypes to identify studies of interest

There is a wealth of expression data within recount2 (9); however, with regards to phenotype information, it is only currently possible to filter on information contained within the abstract. Using predicted phenotypes can help to filter out and identify studies of interest to a particular researcher.

For example, we were interested in identifying a cancer study in which sex was not reported. We hoped to assess the effect of including predicted sex in the analysis of these data. To identify such a study, we filtered through cancer studies included in the SRA to identify studies fulfilling the following criteria: expression data (i) predicted to be generated from paired end sequencing, (ii) where sex was not reported in the SRA metadata, (iii) where the data were predicted to have come from a tissue (rather than a cell line), and finally (iv) that had at least 20 samples. Upon applying these filters, we identified three studies within the SRA meeting these criteria.

### Use case #3: Using predicted phenotypes in analyses

Having identified studies of potential interest, we moved forward with study SRP029880 to assess the utility of the phenotypes we predicted (see Methods). This study includes expression data from 54 samples from 18 unique individuals. For each individual, RNA-seq was carried out on a normal colonic epithelial sample (NC), the primary colon cancer epithelium (PC), and a metastatic cancer sampled from the liver (MC). The initial analysis sought to identify gene expression changes that correspond to aggressiveness of colorectal cancer (CRC). To do so, they authors carried out differential gene expression analyses between NC and MC samples (identifying 2,861 significant genes) and between MC and PC samples (identifying 1846 significant genes). Significance was defined such that *P* < 0.001 and *l*ogFC ≥ 2. No covariates were included for analysis. The authors then, in an attempt to remove the effects of the fact that the MC samples were sampled from the liver while the PC and NC samples were sampled from the colon, filtered out ‘liver specific genes (309 genes)’ using the TiGER database (25) from their MC:PC results.

**Figure 5.**
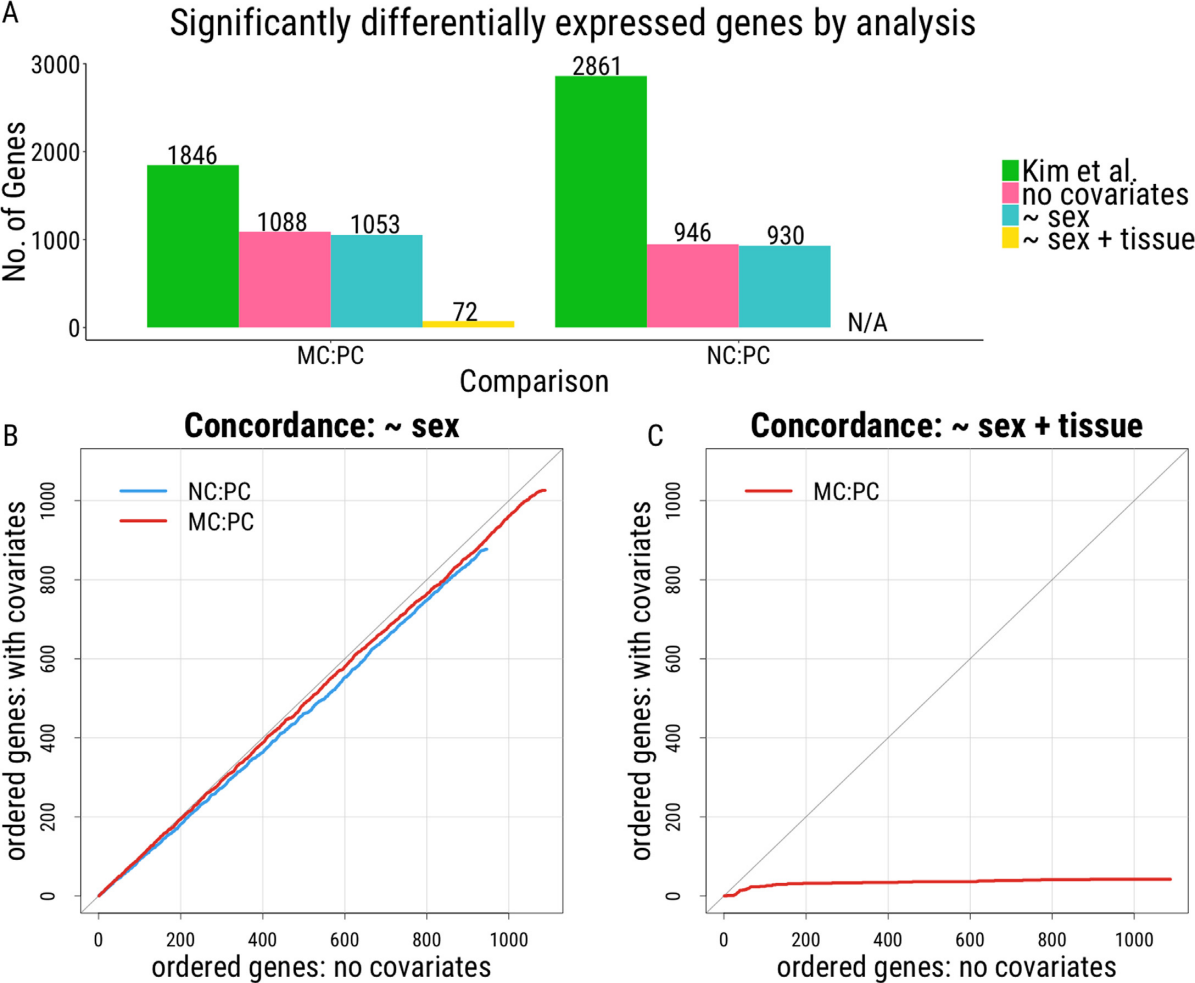
Differential gene expression analysis. (**A**) Number of genes reported significant in Kim *et al.* (23) and the analyses carried out here using their data obtained from recount2. (B, C) Concordance at top (CAT) plots (29) comparing DGEA. The number of genes concordant between analyses are plotted, where perfect agreement between analyses’ results would fall along 45-degree line (gray). DGEA where no covariates were included for analysis (x-axis) were compared to (**B**) DGEA with sex included as a covariate and (**C**) DGEA with both sex and tissue included as covariates. NC = normal colonic tissue; PC = primary colorectal cancer; MC = metastatic cancer (liver).

Here, with prediction data available, we can directly and more appropriately correct for tissue differences between samples and include sex in the differential gene expression analysis (DGEA). Given the experimental design, we checked to ensure that, for each individual, the same sex was predicted across each of the three samples. Predicted sex was concordant across samples within individual for all samples included for study. Analysis was first carried out as in the initial publication (23), comparing NC:PC and MC:PC. We note, however, that expression data utilized herein were obtained from recount2 (9). Thus, rather than using normalized FPKM, as was done in Kim *et al.* (23), expression was instead summarized by scaling the gene counts (see Materials and Methods). These expression estimates were used to carry out DGEA using the same software and cutoffs as in Kim *et al.* (23). Despite using the same software, settings, and thresholds used in Kim *et al.* (23), fewer genes (41.1% and 66.9% less in MC:PC and NC:PC, respectively) were detected to be significantly differentially expressed in our analysis (Figure 5A). As a result, all further comparisons utilized the DGEA results from our analysis, both with and without covariate inclusion to remove the impact of the discrepancy in number of genes identified.

To assess the effects of covariate inclusion on the analysis, DGEA was carried out with either predicted sex or both predicted sex and predicted tissue as covariates. For each of the NC and PC samples, predicted tissue was either ‘colon’ or ‘small intestine.’ Within the MC samples, which were sampled from the liver, four were predicted to be intestinal tissue, the site of the primary lesion, and fourteen to be ‘liver’, the site of the metastases. Interestingly, cancer cell lines do not always recapitulate the tissue from which they were obtained, suggesting that cell lines derived from metastases could, in certain cases, recapitulate the primary tissue from which they were derived (30,31). Here, these findings suggest, unsurprisingly, that the same may be the case at the tissue level. Thus, if the MC samples were all simply labeled ‘liver’, not only would the results change but the analysis would be completely confounded by tissue. In this case, using predicted phenotypes likely allows for a more appropriate differential gene expression analysis to be carried out.

The effects of covariate inclusion were assessed using concordance at the top (CAT) plots (29) comparing concordance between genes identified as significant with and without covariate inclusion. Upon inclusion of sex as a covariate in the analysis, the results remain largely unchanged, where 9 of the 10 most significant genes and 97 of the top 100 genes between analysis are concordant for the MC:PC comparison and 8 of 10 and 91 of 100 genes are concordant for the NC:PC comparison (Figure 5B), suggesting that inclusion of sex had little effect on the results of this analysis. However, upon inclusion of both predicted sex and predicted tissue, the results are dramatically different, with concordance at only 1 of the 10 most significant genes and 24 of the top 100 genes between analyses (Figure 5C). As the MC samples were obtained from a completely different tissue, this finding suggests that many of the differences reported as significant between MC and PC samples simply reflect tissue differences between the samples, rather than differences associated with cancer aggressiveness, despite the removal of ‘liver-specific genes.’

To test this directly, the level of overlap between the aforementioned differential expression analyses and a differential gene expression analysis comparing healthy liver and colon in a completely independent set of samples from the GTEx Project (Supplementary Figure S12A) was assessed. Here, we hypothesized that if the MC:PC comparison is detecting tissue differences, rather than cancer related differences, the comparison between MC:PC differential gene expression and healthy liver and colon tissue from the GTEx data would show more overlap than would the NC:PC comparison to the GTEx data. The results demonstrate that 374 of the top 1000 genes demonstrating differential expression in the GTEx data also are also in the top 1000 most differentially expressed genes in the Kim *et al.* MC:PC comparison, relative to overlap of 31 and 26 of the top 1000 genes in either the NC:PC and MC:PC comparison where tissue has been included in the model, respectively (Supplementary Figure S12B). These results support that, despite removing the ‘liver-specific genes’ (25) from the MC:PC comparison, the differential expression results still reflect tissue differences.

Finally, while inclusion of predictions improves the accuracy of this analysis, we note that the the inclusion of predicted phenotypes in downstream analyses is currently limited, as sex and tissue predictions were made from the expression data, the same data upon which the predictions are modeled as covariates to look for expression differences. Novel statistical approaches will be required to most appropriately utilize these predictions in downstream analyses.

## CONCLUSION

We demonstrate that accurate phenotype prediction from expression data is possible for both biological and technical phenotypes and demonstrate, through three use cases, how predicted phenotypes can be utilized going forward. We have built predictors for sequencing strategy, sex, sample origin, and tissue. These predictors have been applied to the samples within recount2 (9) and can be applied to expression data generated in the future. Predicted phenotypes were generated using the R package phenopredict and can be accessed in the R package recount using the add_predictions() function. We demonstrate how predicted phenotypes can be used to characterize the samples currently within recount2, to identify samples and studies of interest for future study, and how predicted phenotypes can be incorporated into analyses to improve accuracy. Taken together, the availability of this critical phenotype information across the 70,000 samples currently included within recount2 (9) make downstream analyses with these expression data feasible.

## Supplementary Material

Supplementary Data
