# Supplementary material for: Improving the value of public RNA-seq expression data by phenotype prediction
Source: Nucleic Acids Res. 2018 Mar 5;46(9):e54. doi: 10.1093/nar/gky102 (PMC5961118; doi:10.1093/nar/gky102)
Supplement: Supplementary Data [file gky102_supp.zip › nar-02460-met-n-2017-File012.pdf]

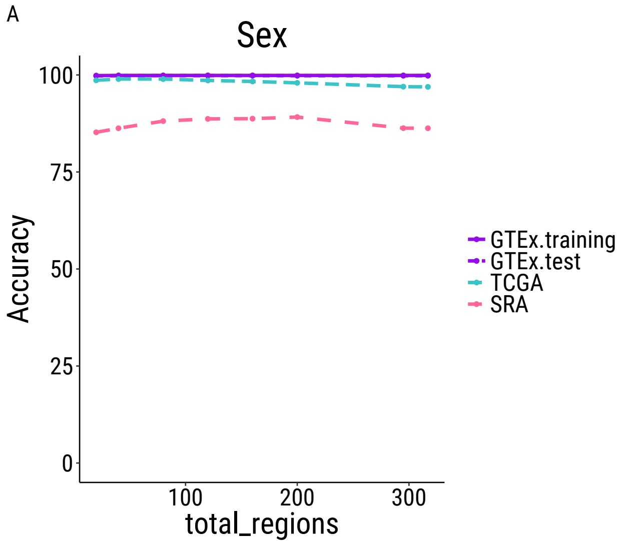

numRegions	10	20	40	60	80	100	150	200
total_regions	20	40	80	120	160	200	295	317
GTEx.training	99.83	99.87	99.87	99.87	99.87	99.87	99.87	99.87
GTEx.test	99.77	99.81	99.83	99.83	99.83	99.81	99.81	99.81
TCGA	98.62	98.95	98.94	98.59	98.32	97.96	96.99	96.93
SRA	85.22	86.26	88.1	88.68	88.74	89.15	86.29	86.26

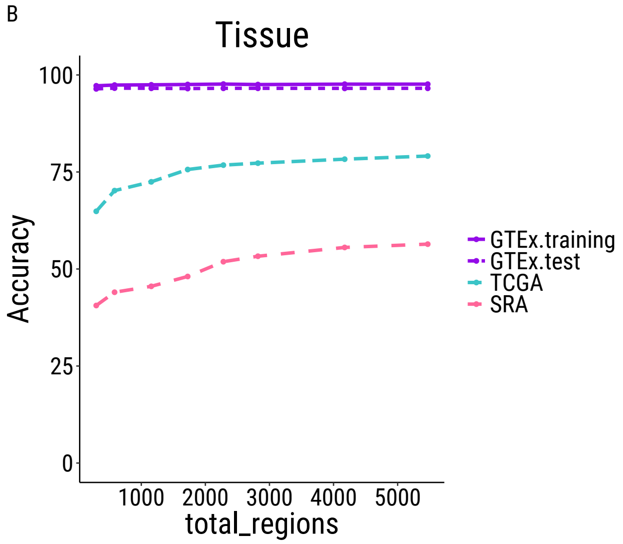

numRegions	10	20	40	60	80	100	150	200
total_regions	297	583	1156	1724	2281	2820	4174	5470
GTEx.training	97.21	97.4	97.46	97.55	97.65	97.53	97.63	97.63
GTEx.test	96.41	96.6	96.56	96.5	96.56	96.56	96.54	96.56
TCGA	64.87	70.2	72.47	75.64	76.77	77.28	78.3	79.11
SRA	40.6	44.02	45.54	48.07	51.88	53.29	55.56	56.41

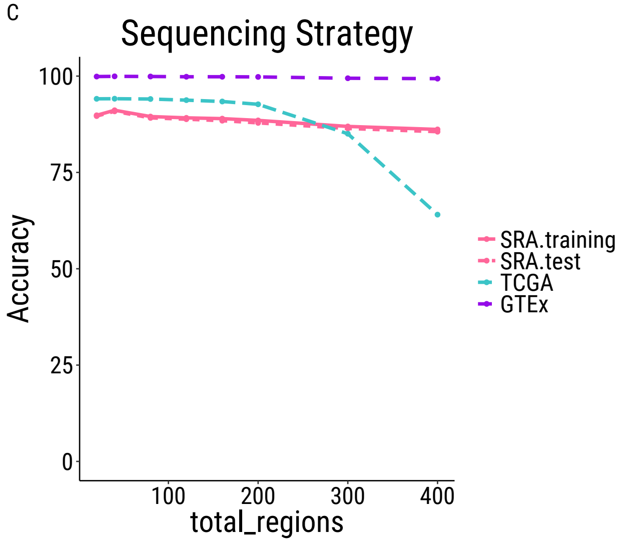

numRegions	10	20	40	60	80	100	150	200
total_regions	20	40	80	120	160	200	300	400
SRA.training	89.83	91.14	89.5	89.17	88.96	88.5	86.94	86.16
SRA.test	89.63	90.86	89.15	88.82	88.43	87.86	86.41	85.57
TCGA	94.11	94.13	94.05	93.76	93.41	92.68	85.04	64.05
GTEx	99.88	99.94	99.9	99.83	99.83	99.79	99.44	99.34

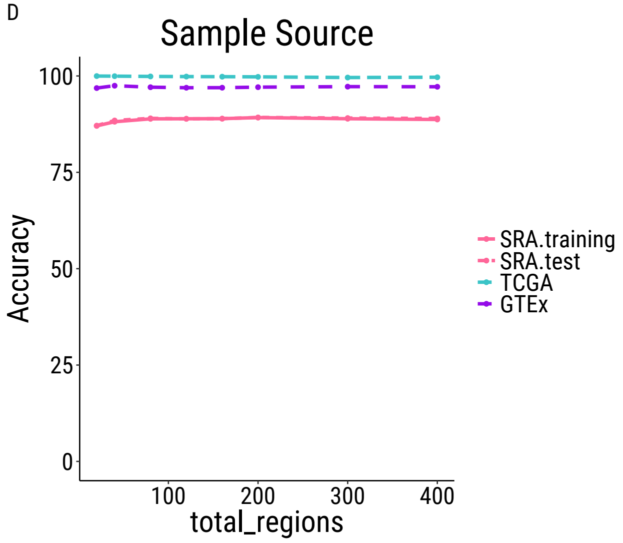

numRegions	10	20	40	60	80	100	150	200
total_regions	20	40	80	120	160	200	300	400
SRA.training	87.05	88.12	88.87	88.89	88.92	89.2	88.9	88.71
SRA.test	87.11	88.45	89.01	88.88	88.93	89.23	89.08	88.98
TCGA	99.97	99.95	99.88	99.84	99.81	99.77	99.59	99.67
GTEx	96.87	97.47	97.09	96.95	96.96	97.1	97.23	97.2

Figure S1: **Region number optimization**. For each phenotype predicted, the relationship between the number of regions used for prediction (x-axis) and prediction accuracy (y-axis) was determined. Optimal region number was decided using the minimum number of regions at which maximum accuracy was achieved in the training set (solid line). Accuracy in the test data sets (dotted lines) are included on graph to illustrate how sensitive each phenotype is to number of regions included. Tables below each figure include the setting for `numRegions` in `build_predictor()`, how many total regions to which this value corresponds `total_regions`, and the prediction accuracy each data set across the various region numbers tested. GTEx data are in purple, TCGA in pink, and SRA in teal.

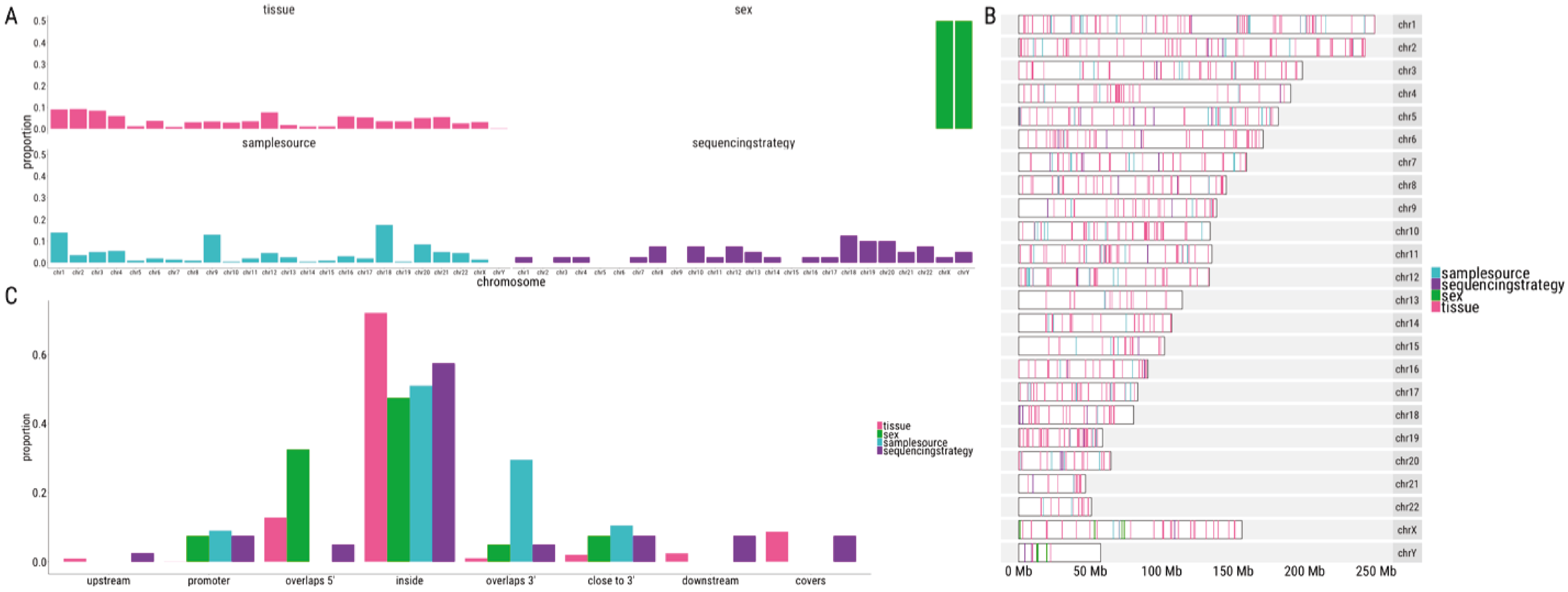

Figure S2: **Prediction region summary**. Regions used for each phenotype predicted are summarized. **A**. The proportion of regions from each chromosome are plotted. **B**. The distribution of regions across each chromosome is plotted. **C**. Each region was annotated with its genomic location. The proportion of regions in each category is plotted for each predicted phenotype. Across all panels, tissue is in pink, sex in green, sample source in teal, and sequencing strategy in purple.

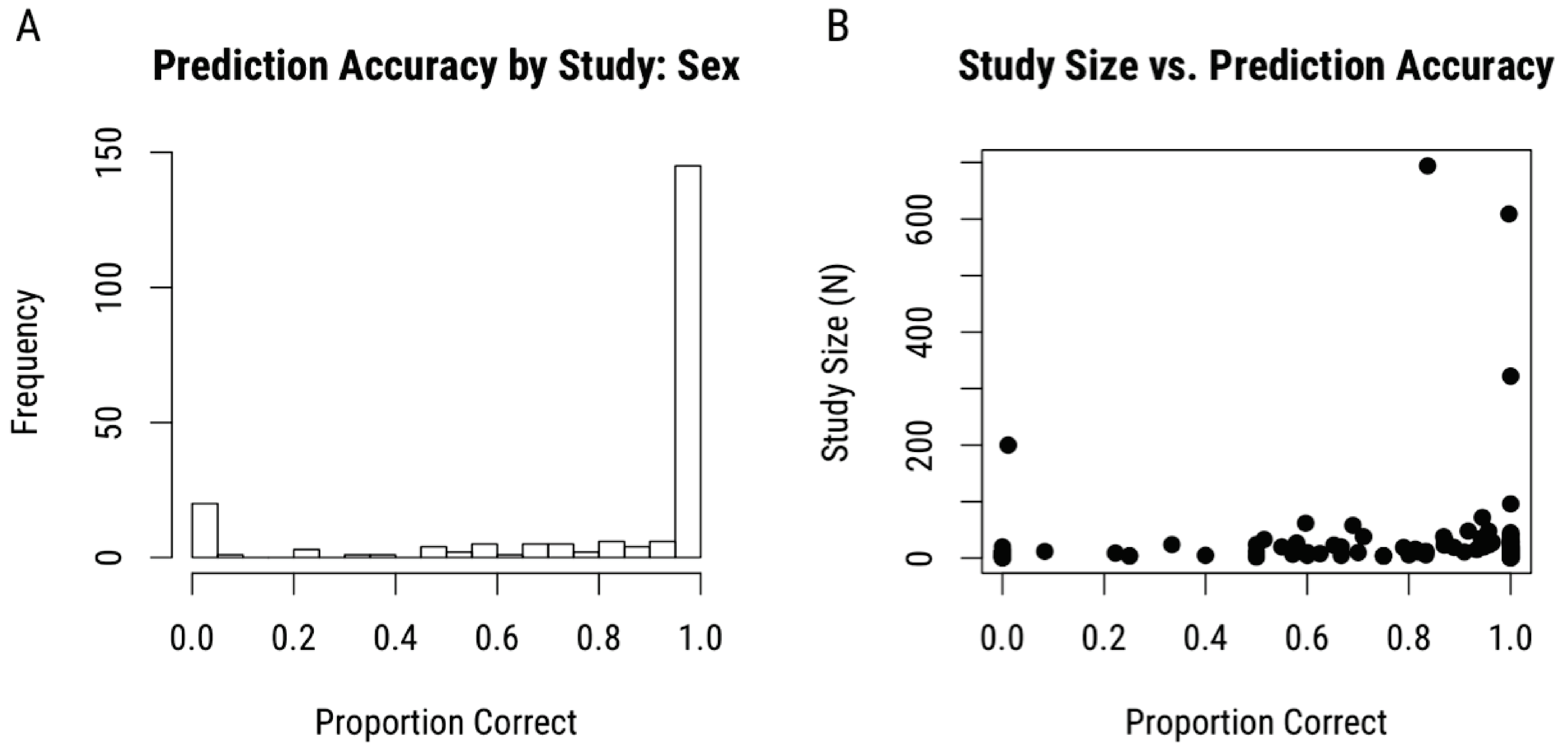

Figure S3: **Study-wise Sex Prediction Accuracy**. **A**. Distribution of study-wise sex prediction accuracies for 211 SRA studies. **B**. Relationship between study sample size and prediction accuracy.

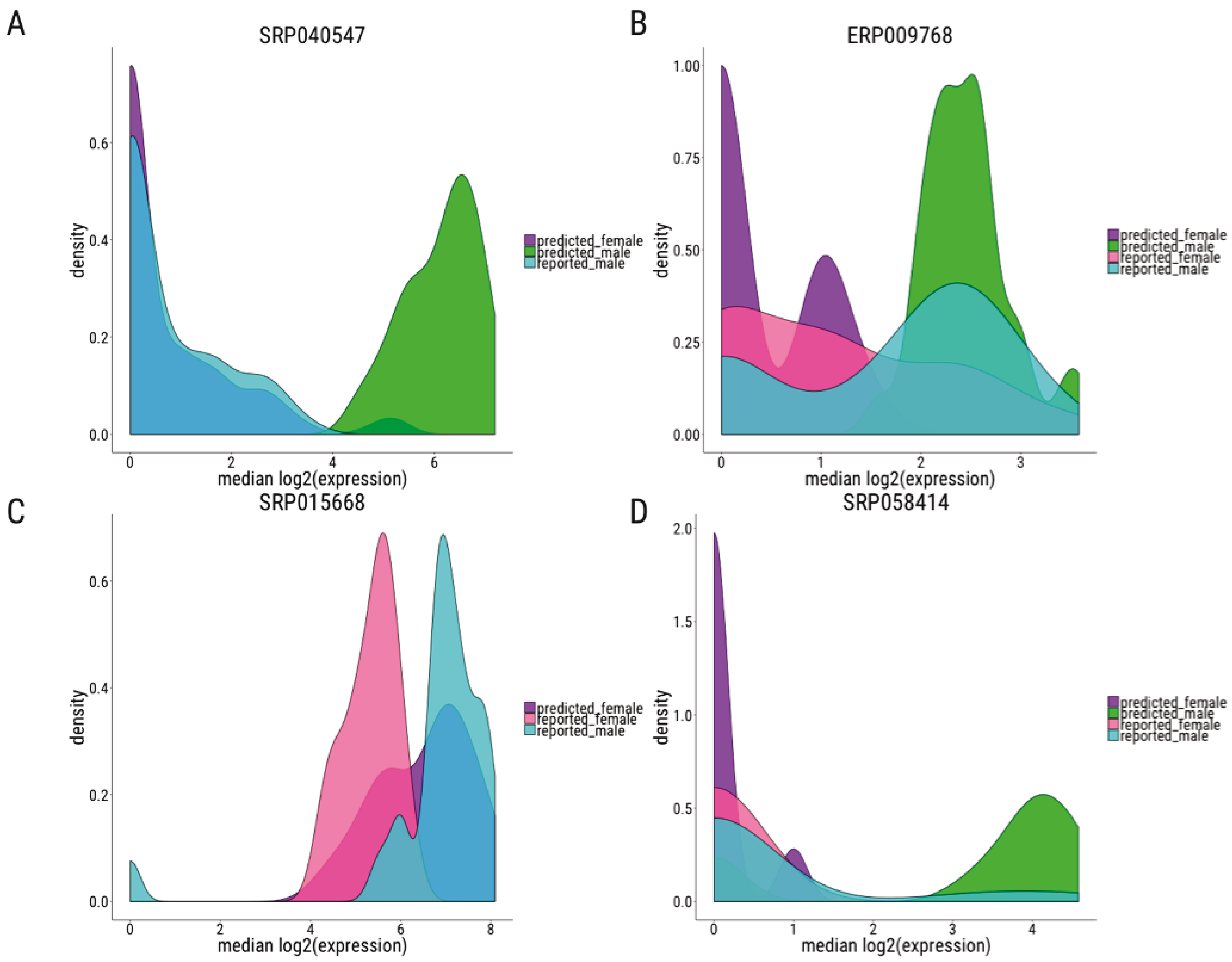

Figure S4: **Median Y chromosome gene expression**. Median expression across highly expressed genes on the Y chromosome for four SRA studies with low sex prediction accuracy. **A**. samples reported to be male in SRP040547 (N=200) show little expression across the Y chromosome, suggesting female samples were mislabeled as male. **B**. ChrY analysis from ERP009768 (N=58) suggests that many samples were likely reported correctly; however, there are likely both male and female samples that were misreported. **C**. SRP015668 (N=33) demonstrates expression from the Y from both samples predicted to be female and reported to be male. Samples reported to be male do have higher expression than those reported to be female, suggesting some likely error in sex prediction. **D**. SRP058414 (N=27) demonstrates that while females were likely reported correctly, a number of samples reported to be male are truly female samples and were thus misreported in the SRA metadata.

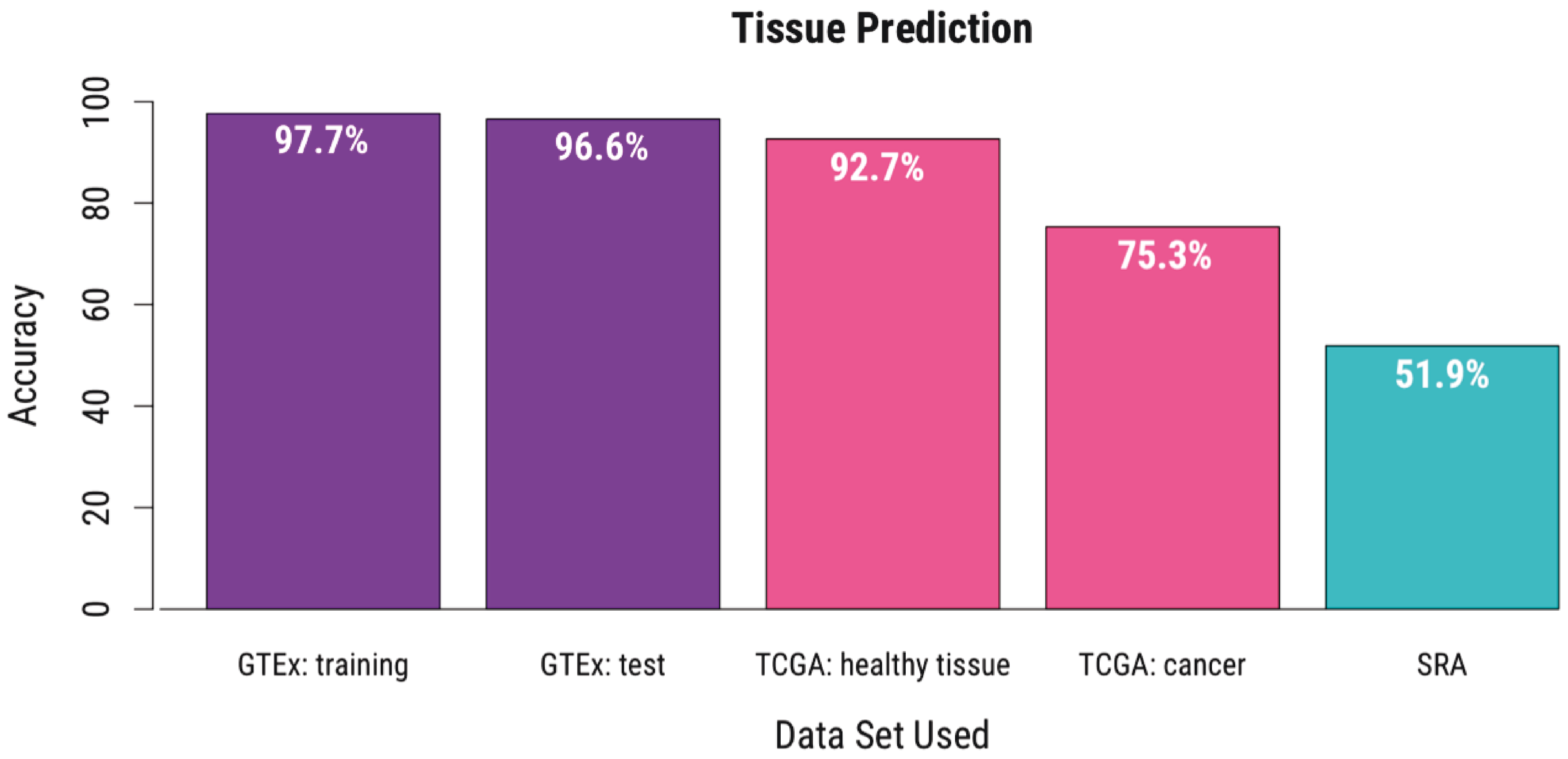

Figure S5: **Tissue prediction within TCGA.** Prediction accuracy within TCGA (pink) shows marked improvement within healthy tissue relative to cancer tissue samples. GTEx data are in purple, TCGA in pink, and SRA in teal.

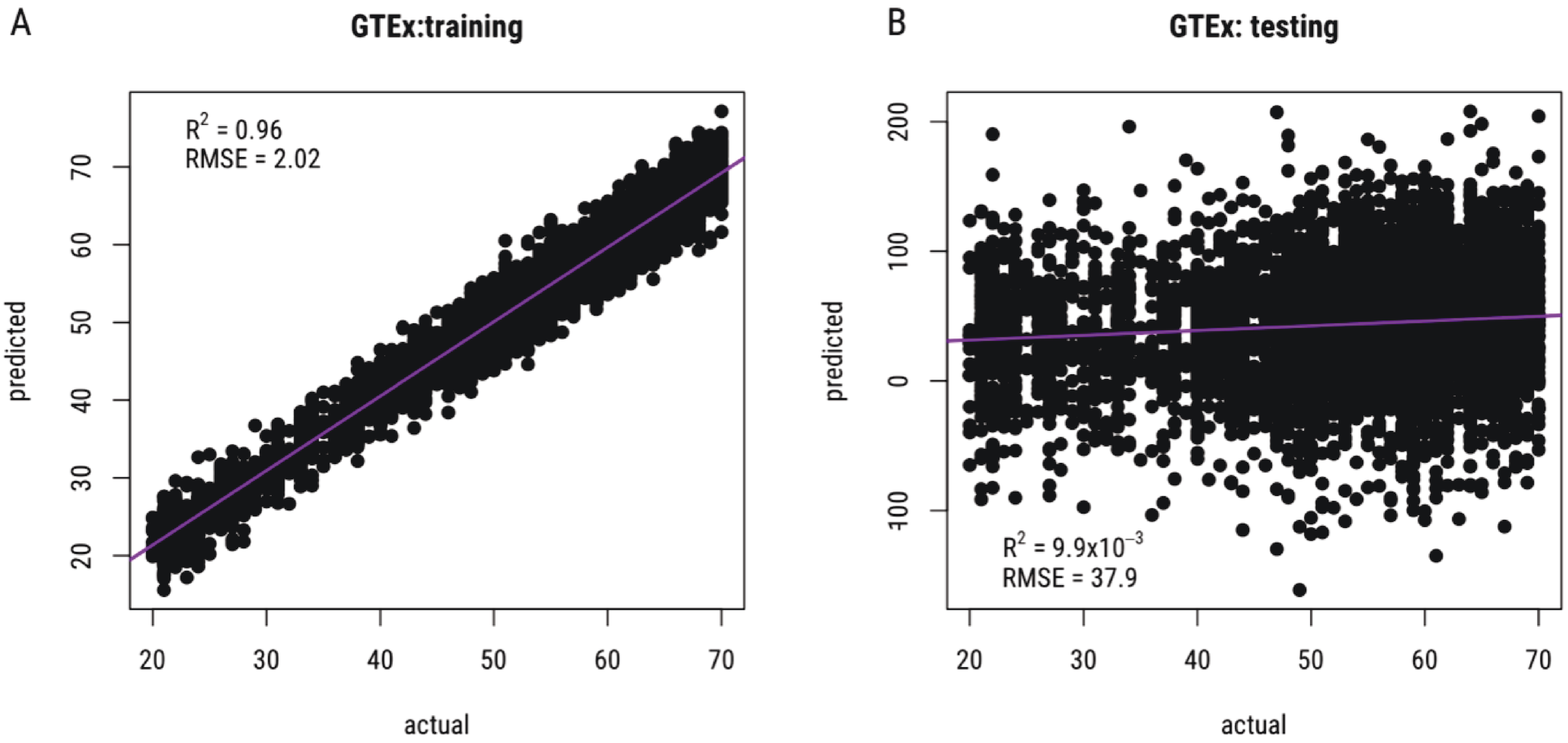

Figure S6: **Age prediction.** Expression data from 900 regions were used to predict age. Predicted ages vs. actual (reported) age (in years) are shown in the **A**. training and **B**. test data sets. Correlation (R^2) and root mean squared error (RMSE) are shown for each data set.

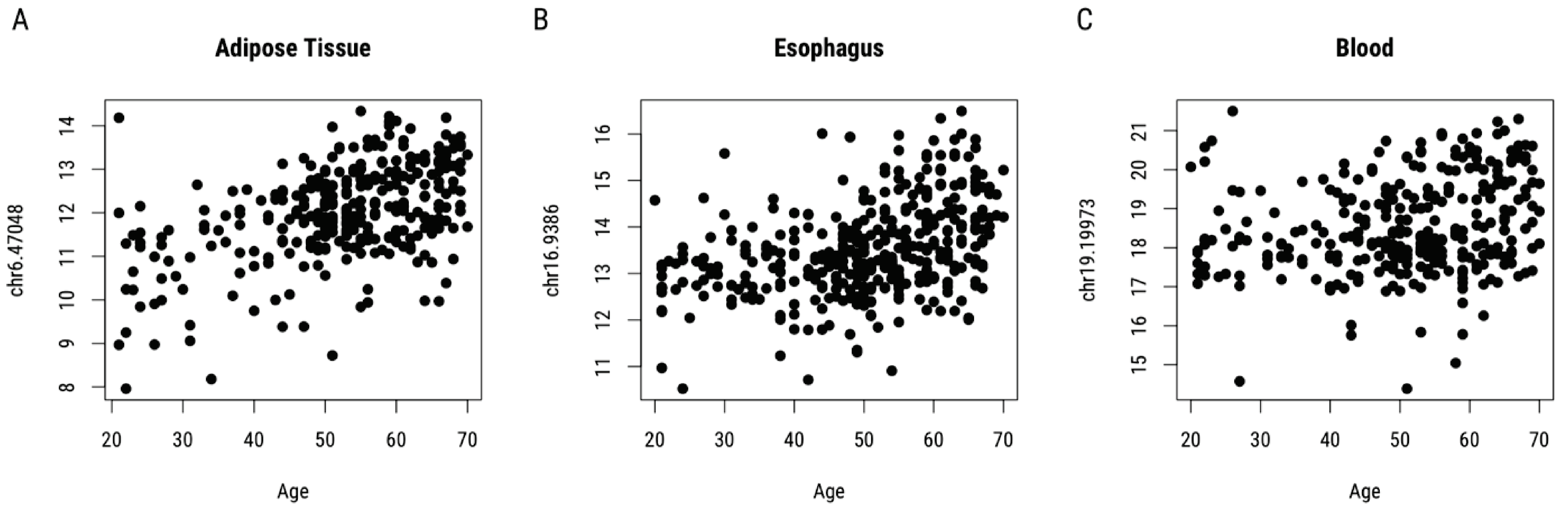

Figure S7: **Expression within tissue.** Expression estimates (log2 scale) for the region most highly associated with age (years) in **A**. adipose tissue **B**. esophagus and **C**. blood are shown.

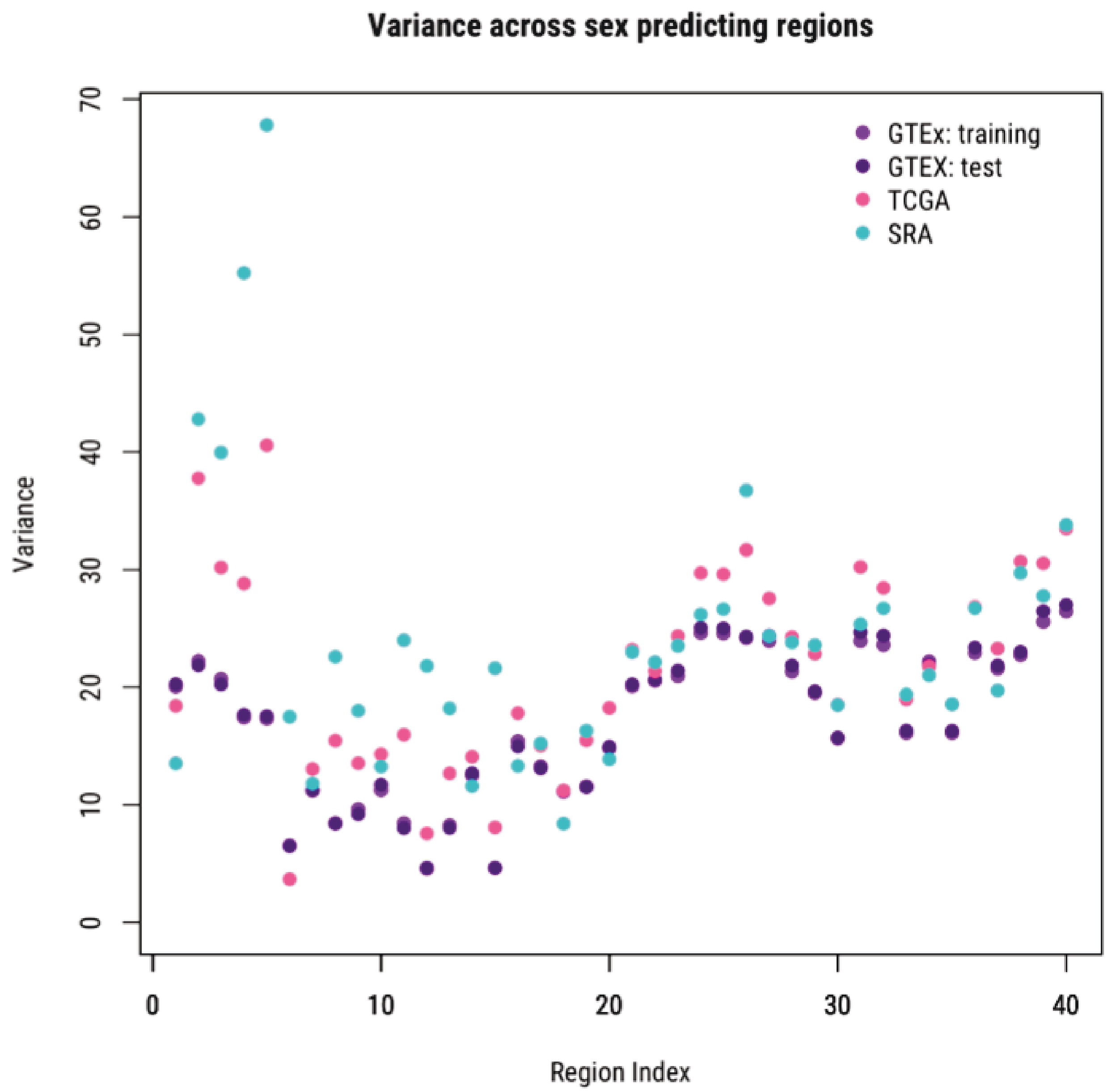

Figure S8: **Variance across sex prediction regions.** Variance in log2 expression at the 40 regions used to predict sex are shown across the data sets within `recount2`. GTEx training and test sets are in purple, TCGA in pink, and SRA in teal. Indices 1-20 correspond to regions on the X chromosome, while 21-40 correspond to the Y chromosome.

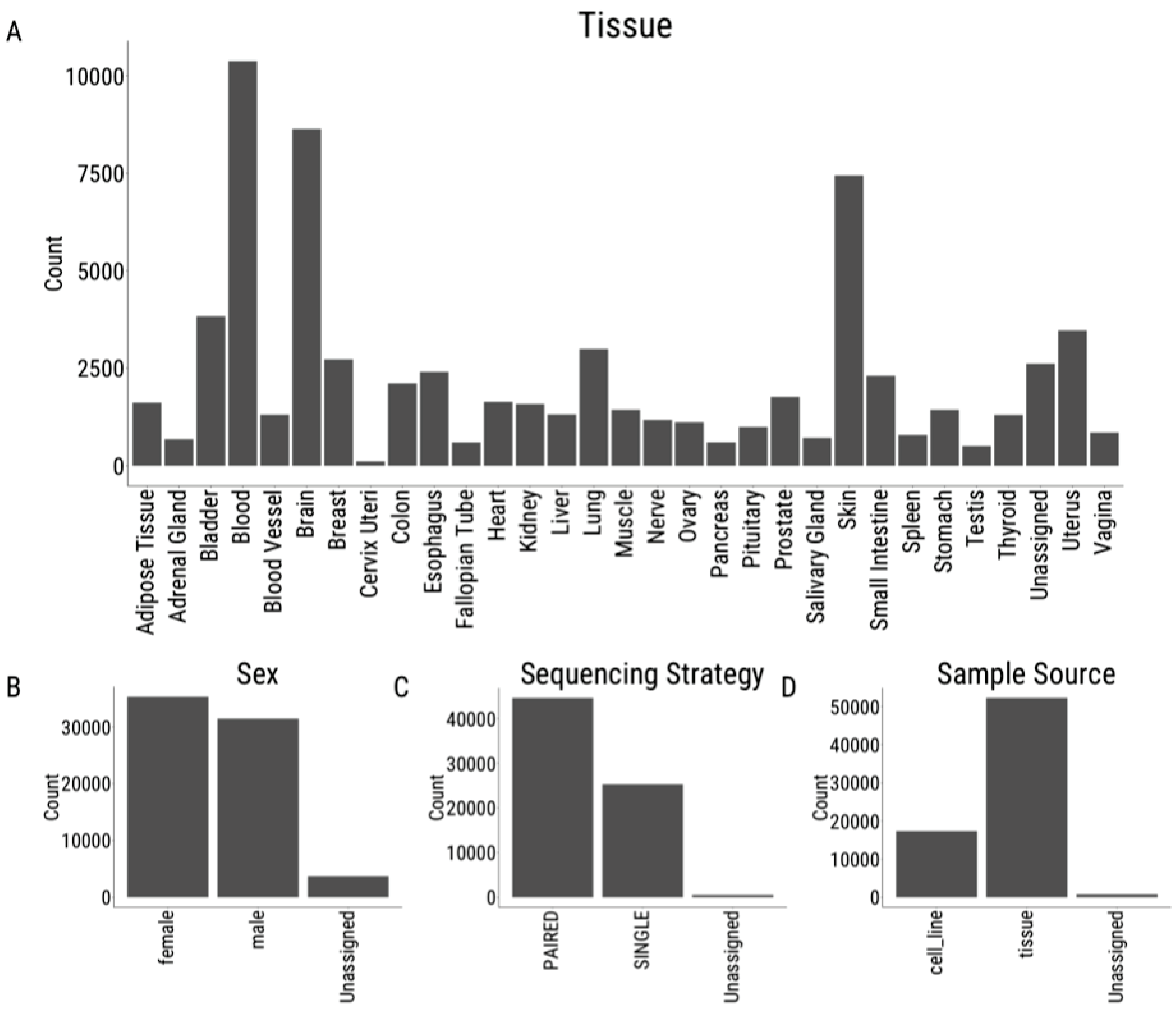

Figure S9: **Predicted phenotypes' relative frequencies**. Prediction frequencies are plotted for **A**. tissue, **B**. sex, **C**.sequencing strategy, and **D**. sample source.

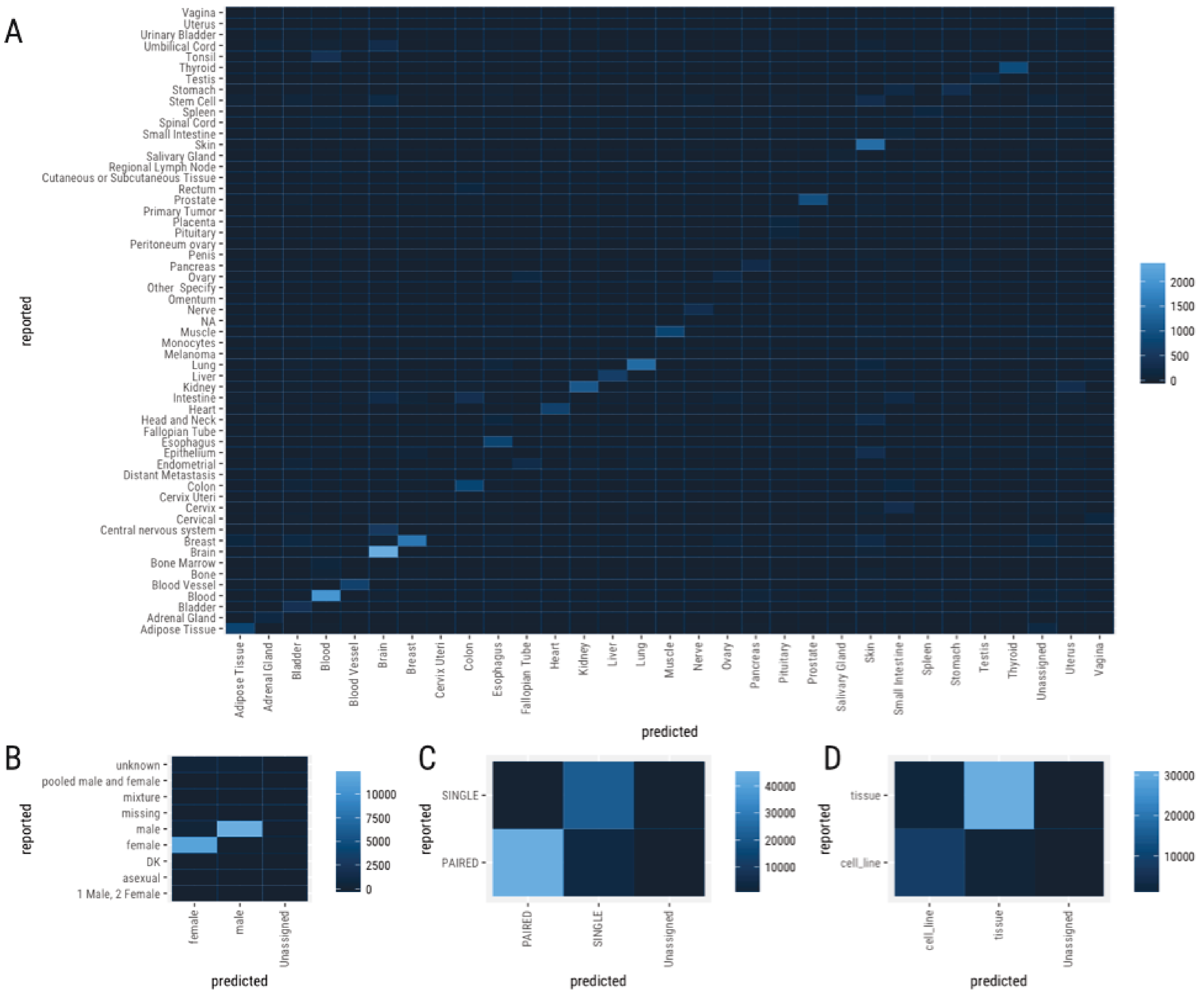

Figure S10: **Confusion matrices**. Confusion matrices for **A**. tissue, **B**. sex, **C**.sequencing strategy, and **D**. sample source. Proportion of samples whose predicted phenotype matches each reported category is plotted.

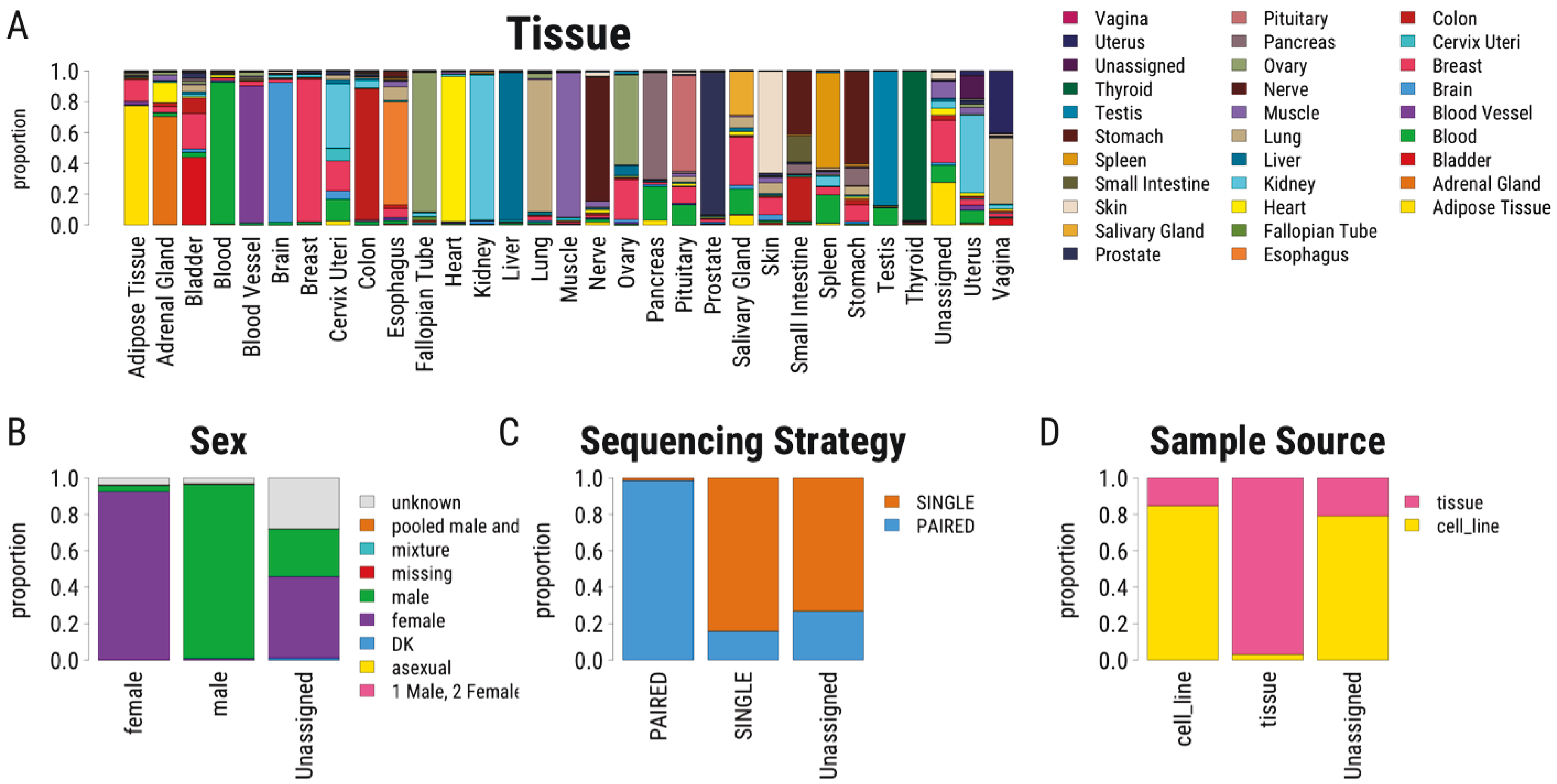

Figure S11: **Relative accuracy for each predictor**. For each phenotype predicted, the proportion of reported phenotypes is displayed for **A**. tissue, **B**. sex, **C**.sequencing strategy, and **D**. sample source.

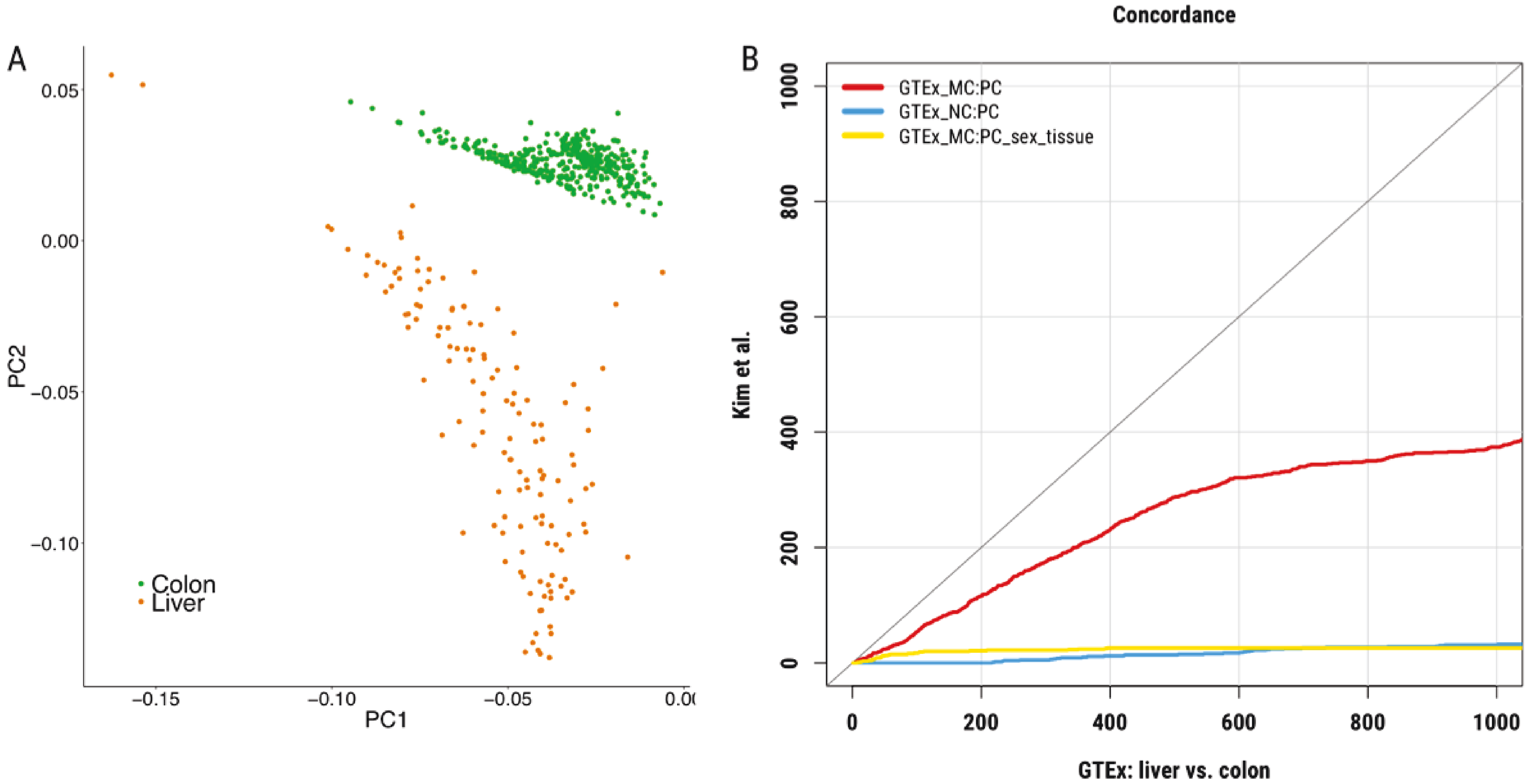

Figure S12: **DGEA comparison to GTEx data**. **A**. PCA plot for GTEx samples used in analysis demonstrate that global gene expression differs between the colon and the liver. Colon samples are in green. Liver samples in orange. **B**. Concordance at the top (CAT) plot for overlap between the top 1000 differentially expressed genes in the GTEx samples (liver vs. colon) and the DGEA results from the Kim et. al expression data. Perfect agreement between analyses' results would fall along 45-degree line (grey). Comparison between GTEx results and the metastatic liver and primary colon samples is in red. Comparison to metastatic liver and primary colon samples, corrected for sex and tissue, is in yellow. Comparison to primary and healthy colon samples is in blue.
